# Dynamic Associations Between Centers for Disease Control and Prevention Social Media Contents and Epidemic Measures During COVID-19: Infoveillance Study

**DOI:** 10.2196/49756

**Published:** 2024-01-23

**Authors:** Shuhua Yin, Shi Chen, Yaorong Ge

**Affiliations:** 1 University of North Carolina at Charlotte Charlotte, NC United States

**Keywords:** infoveillance, social media, COVID-19, US Centers for Disease Control and Prevention, CDC, topic modeling, multivariate time series analysis

## Abstract

**Background:**

Health agencies have been widely adopting social media to disseminate important information, educate the public on emerging health issues, and understand public opinions. The Centers for Disease Control and Prevention (CDC) widely used social media platforms during the COVID-19 pandemic to communicate with the public and mitigate the disease in the United States. It is crucial to understand the relationships between the CDC’s social media communications and the actual epidemic metrics to improve public health agencies’ communication strategies during health emergencies.

**Objective:**

This study aimed to identify key topics in tweets posted by the CDC during the pandemic, investigate the temporal dynamics between these key topics and the actual COVID-19 epidemic measures, and make recommendations for the CDC’s digital health communication strategies for future health emergencies.

**Methods:**

Two types of data were collected: (1) a total of 17,524 COVID-19–related English tweets posted by the CDC between December 7, 2019, and January 15, 2022, and (2) COVID-19 epidemic measures in the United States from the public GitHub repository of Johns Hopkins University from January 2020 to July 2022. Latent Dirichlet allocation topic modeling was applied to identify key topics from all COVID-19–related tweets posted by the CDC, and the final topics were determined by domain experts. Various multivariate time series analysis techniques were applied between each of the identified key topics and actual COVID-19 epidemic measures to quantify the dynamic associations between these 2 types of time series data.

**Results:**

Four major topics from the CDC’s COVID-19 tweets were identified: (1) information on the prevention of health outcomes of COVID-19; (2) pediatric intervention and family safety; (3) updates of the epidemic situation of COVID-19; and (4) research and community engagement to curb COVID-19. Multivariate analyses showed that there were significant variabilities of progression between the CDC’s topics and the actual COVID-19 epidemic measures. Some CDC topics showed substantial associations with the COVID-19 measures over different time spans throughout the pandemic, expressing similar temporal dynamics between these 2 types of time series data.

**Conclusions:**

Our study is the first to comprehensively investigate the dynamic associations between topics discussed by the CDC on Twitter and the COVID-19 epidemic measures in the United States. We identified 4 major topic themes via topic modeling and explored how each of these topics was associated with each major epidemic measure by performing various multivariate time series analyses. We recommend that it is critical for public health agencies, such as the CDC, to update and disseminate timely and accurate information to the public and align major topics with key epidemic measures over time. We suggest that social media can help public health agencies to inform the public on health emergencies and to mitigate them effectively.

## Introduction

The COVID-19 pandemic caused more than 760 million cases and 6.8 million deaths globally as of April 2023 [1]. Therefore, it is crucial for public health agencies, such as the US Centers for Disease Control and Prevention (CDC), to quickly and effectively disseminate up-to-date and reliable health information to the public to curb the pandemic. Over the past years, social media has been widely used by various public health agencies to make announcements, disseminate information, and deliver guidelines of effective interventions to the public. The CDC is among the early adopters of social media to engage with the public, increase health literacy in the society, and promote healthy behaviors [2]. Moreover, the CDC’s social media team has developed the Health Communicator’s Social Media Toolkit to efficiently use social media platforms; map health strategies; listen to health concerns from the public; and deliver evidence-based, credible, and timely health communications in multiple formats such as texts, images, and videos. The CDC’s digital health communication efforts have been especially established on various social media platforms such as Twitter, Facebook, and Instagram.

Building successful interactions with the public relies on people understanding the content and raising awareness of it. The CDC has been heavily engaging in social media presence [3]. For example, during the COVID-19 pandemic since 2019, it has been responsive and proactive on Twitter to continuously tweet about reliable health-related messages and quickly diffuse public engagement by responding to user comments, retweeting credible sources, and monitoring online conversations in real time. Hence, it is meaningful to recognize the COVID-19 pandemic information disseminated by the CDC on social media, characterize various contents and topics, and evaluate posting patterns with regard to the actual epidemic dynamics. Monitoring the content, topics, and trends will help identify current issues or interests and the levels of interventions. It is critical to evaluate the associations between various COVID-19 content topics tweeted by the CDC and the actual COVID-19 epidemic measures (eg, cases, deaths, testing, and vaccination records). Knowing the underlying associations between the CDC’s digital health communication contents on social media and the actual COVID-19 epidemics will help in understanding and evaluating the CDC’s tweeting patterns with changes in the epidemic, and will further help in recommending more effective social media communication strategies for public health agencies accordingly.

Infodemiology and infoveillance studies tackle health challenges, generate insights, and predict patterns and trends of diseases using previously neglected online data. Infodemiology, which is the conjunction of “information” and “epidemiology,” defined by Gunther Eysenbach, is the field of distribution and determinants of information of a population through the internet or other electronic media [4]. Infoveillance takes surveillance as the primary aim and generates automated analysis from massive online data. It employs innovative computational approaches to mine and analyze unstructured online text information, such as analyzing patterns and trends, predicting potential outbreaks, and addressing current issues of public health. Unlike traditional epidemiological surveillance systems, which include cohort studies, disease registries, population surveys, and health care records, infoveillance studies discover a wide range of health topics, monitor health issues including outbreaks and pandemics, and forecast epidemiological trends in real time. A large amount of anonymous online data can be obtained in a more timely manner with these approaches than with traditional surveillance systems, and this will help researchers and public health agencies to prepare for and tackle public health emergencies and issues more efficiently and effectively.

Social media platforms have been having impacts on the community education of COVID-19 and delivering various health information about the disease. Many studies have also incorporated the concept of infoveillance by analyzing unstructured textual data obtained from social media. Liu et al [5] collected and analyzed media reports and news articles on COVID-19 to derive topics and useful information. They aimed to investigate the relationship between media reports and the COVID-19 outbreak, and the patterns of health communication on the coronavirus through mass media to the general audience. They obtained media reports and articles related to the pandemic and studied prevalent topics. There had been prevalent public discussions of attitudes and perspectives on mask-wearing on social media. Therefore, it is important for public health agencies to disseminate the supporting evidence and benefits of masking to mitigate the spread of COVID-19. Al-Ramahi et al [6] studied the topics associated with the public discourse against wearing masks in the United States on Twitter. They identified and categorized different topics in their models. These studies all applied infoveillance to investigate the potential impacts of diseases, health behaviors, or interventions on target populations, communities, and the society. However, mass media and social media are also prone to the spreading of misinformation and conspiracy theories, especially from unreliable sources [7]. Hence, the sources of information obtained from social media are crucial as misinformation could potentially create bias, mislead public perceptions, and provoke negative emotions. Official accounts of public health agencies are usually sources of unbiased and reliable health information. Although there have been several studies that collectively explored the topics discussed by the general public on social media during the pandemic, no investigations have been performed so far to identify various topics from health agencies, such as the CDC, during a large health emergency.

Furthermore, information discrepancies and delays could occur between topics posted by health agencies and real-time epidemic trends. Such discrepancies could cause confusion among the public on interventions for health emergencies. Therefore, quantifying their associations is important to reduce knowledge gaps. Chen et al [8] studied correlations between the Zika epidemic in 2016 and the CDC’s responses on Twitter. They quantified the association between the 2 types of data through multivariate time series analyses and information theory measurements. The study discovered the CDC’s varying degrees of efforts in disseminating health-related information to the public during different phases of the Zika pandemic in 2016. However, no study so far has investigated such dynamic associations, more specifically, the CDC’s COVID-19 content topic tweeting patterns and the actual COVID-19 epidemic metrics.

While still being investigated, it is imperative to understand the dynamic associations between various content topics on social media and actual epidemic outcome metrics, which will guide health agencies to identify driving factors between the 2 and help in disseminating helpful knowledge to the public accordingly. In this study, we aimed to discover the underlying COVID-related topics posted by the CDC during different phases of the COVID-19 pandemic. We also aimed to further quantify and evaluate the dynamic associations between content topics of the pandemic and multiple COVID-19 epidemic metrics. The findings of this study will significantly increase our knowledge about the efficiency of the CDC’s health communications during the pandemic and help make further recommendations for the CDC’s social media communication strategies with the public in the future.

## Methods

### Data Acquisition and Preprocessing

Using the Twitter academic API (application programming interface) and search query (see search query in Multimedia Appendix 1), we retrieved a total of 17,524 English tweets posted by 7 official CDC-affiliated Twitter accounts up to January 15, 2022 (for the detailed acquisition process for CDC tweets, see Multimedia Appendix 1). We also acquired the COVID-19 epidemic metric data in the United States from the Johns Hopkins University – Center for Systems Science and Engineering (CSSE) public GitHub repository [9-11]. Four sets of important COVID-19 time series data were retrieved, including daily cumulative confirmed cases, deaths, testing, and vaccination. The data were all at the US national level. The 4 sets of original COVID-19 time series data consisted of dates and their cumulative targeted measurements. The case series set included the daily cumulative number of confirmed COVID-19 reported cases, and it had 751 records, ranging from January 22, 2020, to February 10, 2022. The death series set reported the daily cumulative number of confirmed COVID-19 death cases, and it had 908 records, ranging from January 22, 2020, to July 17, 2022. The testing data set reported the daily cumulative number of completed polymerase chain reaction (PCR) tests or other approved nucleic acid amplification tests, and it had 760 records, ranging from January 13, 2020, to February 10, 2022. The vaccination data set included the daily cumulative number of people who received a complete primary series of vaccine doses from the CDC Vaccine Tracker, and it had 428 records, ranging from December 10, 2020, to February 10, 2022.

For consistency in subsequent analyses, all CDC tweet time series and US COVID-19 variable time series were standardized to the same time span in this study, ranging from the start date of reported case data (January 22, 2020) to the end date of CDC tweet collection (January 15, 2022), with a total of 725 records for each data type. Since vaccination data were not available until late 2020, missing values were filled with zeros. In summary, we had 4 time series from 4 different COVID-19 US epidemic metrics and another time series of number of tweets from all 7 CDC-associated Twitter accounts.

### Natural Language Processing

In order to identify major topics in the CDC’s COVID-19 tweets, we performed various natural language processing (NLP) steps. NLP, especially topic modeling, provides granular characterization of textual inputs such as the CDC’s COVID-19 communications.

Regular expressions were first applied to process tweet texts by removing @mentions, hashtags, special characters, emails, punctuations, URLs, and hyperlinks. Tokenization was performed to break down sentences into individual tokens, which can be individual words or punctuations. For example, the sentence “As COVID19 continues to spread, we must remain vigilant” becomes tokens of “*As*,” “*COVID19*,” “*continues*,” “*to*,” “*spread*,” “*,*,” “*we*,” “*must*,” “*remain*,” and “*vigilant*” after tokenization. Next, lemmatization, a structural transformation where each word or token is turned to its base or dictionary form of the morphological information, was performed. For example, for words “studies” and “studying,” the base form, or lemma, was the same “study.” In addition to stop word removal via the Python NLTK library, we created our own list of stop words and removed them from the texts (see the stop words list in Multimedia Appendix 1). With help from domain experts, we excluded stop words that did not contribute to topic mapping.

N-grams, phrases with *n* words, were developed with a threshold value of 1 to form phrases from tweets. Phrase-level *n*-grams were applied here because phrases offer more semantic information than individual words [12]. A higher threshold value resulted in fewer phrases to be formed. The texts were mapped into a dictionary of word representations, which was a list of unique words, and it was then used to create bag-of-words presentations of the texts. A term frequency-inverse document frequency (TF-IDF) model was implemented to evaluate the importance and relevancy of the words to a document. It was calculated by multiplying term frequency, which is the relative frequency of a word within a document, with inverse document frequency, which measures how common or rare a word is across a corpus. A higher TF-IDF value indicates that the word is more relevant to the document it is in [13,14]. Words that were missing and lower than the threshold value of 0.005 from the TF-IDF model were excluded. Table 1 shows the process of data collection and preprocessing, and Table 2 shows the steps of subsequent NLP and statistical analyses.

**Table 1:** Data collection and preprocessing.

Variable	Data collection	Data preprocessing
CDC^a^ tweets	Twitter API^b^ using a search query 17,524 English tweets by January 15, 2022	Remove @mentions, hashtags, special characters, emails, punctuations, URLs, and hyperlinks Tokenization: break down sentences into individual tokens Lemmatization: each word or token is turned to its base or dictionary form Remove a list of stop words created by research experts N-grams: form phrases from the tweets Modify the date range: January 22, 2020 (the start date of reported case data) to January 15, 2022 (the end date of CDC tweets)
COVID-19 epidemic metrics	Public GitHub repository of the CSSE^c^ at Johns Hopkins University Confirmed case count: 751 records; January 22, 2020, to February 10, 2022 Death count: 908 records; January 22, 2020, to July 17, 2022 Completed COVID-19 tests: 760 records; January 13, 2020, to February 10, 2022 Complete vaccination: 428 records; December 10, 2020, to February 10, 2022	Standardize metric time series to be the same as that of CDC tweets Fill missing values in the vaccination data with zeros 725 records for each of the 4 metric series Turn cumulative records to daily records

^a^CDC: Centers for Disease Control and Prevention.

^b^API: application programming interface.

^c^CSSE: Center for Systems Science and Engineering.

**Table 2:** Subsequent analyses.

Variable	Topic modeling	Data analysis
CDC^a^ tweets and COVID-19 metrics	Construct an LDA^b^ topic model using CDC tweets assigning 4 topics Extract generated topics with their top 10 unique associated keywords Produce interactive visualizations using pyLDAvis	Domain experts examine topic keywords with randomly sampled tweets in iteration Domain experts determine the theme of each topic Perform multivariate time series analyses between each topic time series and each COVID-19 metric time series: 1. Visualization 2. Cross-correlation function (CCF) 3. Mutual information (MI) 4. Autoregressive integrated moving average with external variable (ARIMAX) model

^a^CDC: Centers for Disease Control and Prevention.

^b^LDA: latent Dirichlet allocation.

### Topic Modeling With Latent Dirichlet Allocation

To identify more specific topics from all the COVID-19 tweets posted by the CDC, we performed topic modeling via latent Dirichlet allocation (LDA). LDA automatically generates nonoverlapping clusters of words (ie, clusters of words based on their distributions in their corresponding topics) that represent different topics based on probabilistic distributions across the whole corpus (ie, all CDC tweets in this study). LDA was developed to find latent, hidden topics from a collection of unstructured documents or a corpus with text data. Topic models are probabilistic models that perform at 3 levels of documents: a word, a document, and a corpus consisting of multiple documents. The details of LDA and topic models are provided in Multimedia Appendix 1. We investigated and compared across 3 to 8 potential topics and determined the optimal number of topics based on both topic model evaluation and domain expert interpretations of the identified topic clusters.

Model perplexity and topic coherence scores were calculated as performance metrics of LDA. Perplexity is a decreasing “held-out log-likelihood” function that assesses LDA performance using a set of training documents. The trained LDA model is then used to test documents (held-out set). The perplexity of a probability model *q* on how well it predicts a set of samples x*_1_*, x*_2_*, ..., x*_N_* drawn from an unknown probability distribution *p*, is defined as follows [15]:



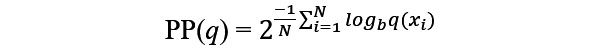



An ideal *q* should have high probabilities q(x*_i_*) for the new data. Perplexity decreases as the likelihood of the words in new data increases. Therefore, lower perplexity indicates better predictability of an LDA model.

Topic coherence assesses the quality of the topics, which is measured as the understandability and semantic similarities between high scoring words (ie, the words that have a high probability of occurring within a particular topic) in topics generated by LDA [16]. We used the UMass coherence score [17], which accounts for the order of a word appearing among the top words in a topic. It is defined as follows [18]:



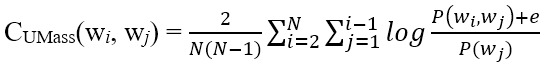



where *N* is the number of top words of a topic of a sliding window, *P*(w*_i_*) is the probability of the *i*th word w appearing in the sliding window that moves over a corpus to form documents, and *P*(w*_i_*, w*_j_*) is the probability of words w*_i_* and w*_j_* appearing together in the sliding window. According to the study from UMass, coherence decreases initially and becomes stationary as the number of topics increases [16].

Representations of all topics were presented in word-probability pairs for the most relevant words grouped by the topics. Interactive visualizations were produced using the pyLDAvis package in Python 3.7 to examine the topics generated by LDA and their respective associated keywords. A data frame of all dominant key topics was created. The original unprocessed full texts of the CDC tweets, IDs, and posting dates were combined into a data frame along with their corresponding key topic number labels and topic keywords. In addition, the daily percentage of each topic from LDA was calculated for further time series analysis. For instance, vaccine/vaccination is an identified key topic, so the percentage of vaccine-related CDC tweets on each day was calculated for the entire study period to construct the vaccine/vaccination-specific topic time series. Since LDA is technically an unsupervised clustering method, after the topics or clusters of word distributions from the CDC’s tweets were generated using LDA, domain experts were involved to further label and interpret the content of the topics using domain knowledge. We randomly generated 20 sample tweets from each topic using Python for domain experts to examine, analyze, and determine the themes of the topics. For each topic, LDA provided a list of the top keywords associated with that topic, and we selected the top 10 keywords. We examined these keywords and referred to the 20 sample tweets, and then derived a theme or context that encompasses these keywords and the original tweets through further discussions, which was important for understanding the context in which these words were used. The final agreement on the interpretation of LDA-generated topics was reached after multiple iterations and discussions of the above process.

### Multivariate Time Series Analyses Between Identified CDC Tweet Topics and COVID-19 Epidemic Metrics

#### Data Preparation

Key topic time series data were derived from the previous NLP and LDA processes. We constructed a multivariate data frame with posting dates and number of tweets for each key topic at a daily resolution. Since LDA identified 4 key topics, a total of 4 CDC key topic time series were developed. There were also 4 US COVID-19 epidemic metric time series: daily cumulative reported cases, cumulative confirmed deaths, cumulative number of completed PCR tests or other approved nucleic acid amplification tests, and cumulative number of people who received a complete primary series of vaccines. These 4 sets of COVID-19 epidemic metric time series were then converted to daily measures via first order differencing. Multivariate time series analyses were implemented to investigate the associations between time series of key CDC tweet topics and US COVID-19 epidemic metrics.

#### Visualizations

Both types of time series, CDC key topics and COVID-19 metrics, were visually inspected in the same plot on double y-axes, with the left y-axis displaying the daily COVID-19 metric and right y-axis displaying the daily CDC tweet topic count. In addition, each plot was further divided based on COVID-19 phases with different dominant variants: the original, Alpha, Delta, and Omicron variants, with their corresponding starting dates: March 11, 2020; December 29, 2020; June 15, 2021; and November 30, 2021, respectively. This helps further observe and identify dynamic changes of time series and their associations during different phases of the pandemic.

#### Cross-Correlation Function

Between 2 time series (also known as signals *x* and *y*), the cross-correlation function (CCF) [19] quantifies their levels of similarities (ie, how similar the 2 series are at different times), their associations (ie, how values in one series can provide information about the other series), and when they occur [20]. The CCF takes the sum of the product for each of the *x* and *y* data points at time lag *l*, defined as follows [19]:



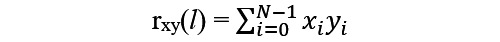



where *N* is the number of observations in each time series, and x*_i_* and y*_i_* are the observations at the *i*th time step in each of the time series. The CCF ranges from −1 to 1, and a larger absolute value of the CCF is related to a greater association shared by the 2 time series at a given time lag *l* [21]. In this study, each of the 4 CDC tweet topic time series was compared with each of the 4 COVID-19 epidemic metric time series to calculate their respective CCFs. All CCF values were calculated with a maximum lag of 30 days, as we assumed that the real-world epidemic could not influence online discussions for more than a month and vice versa.

#### Mutual Information

Mutual information (MI) was calculated by computing the entropy of the empirical probability distribution to further quantify the association between each of the 4 key CDC tweet topics and each of the 4 US COVID-19 epidemic metrics. MI measures the amount of mutual dependence or average dependency between 2 random variables *X* and *Y*. It is defined as follows [22]:



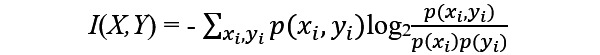



where *x_i_* and *y_i_* are the *i*th elements of the variables *X* and *Y*, respectively. When applied to time series data, *X* and *Y* are 2 individual time series and *x_i_* and *y_i_* are their respective observations at the *i*th time step. Note that MI is a single value instead of a function over lag *l* as in the CCF. A larger MI value indicates a higher shared mutual dependency between the 2 time series.

#### Autoregressive Integrated Moving Average With External Variable

Neither the CCF nor MI differentiate dependent and independent variables, that is, the formula was symmetric with regard to *X* and *Y* variables. We further evaluated whether the CDC tweeting topics were influenced by real-world COVID-19 epidemic outcomes. An autoregressive integrated moving average with external variable (ARIMAX) model was constructed to fit each of the 4 CDC topics with each of the 4 COVID-19 epidemic metrics during the entire study period. A univariate autoregressive integrated moving average (ARIMA) model fits and forecasts time series data with the integration of an autoregressive (AR) component and a moving average (MA) component with their respective orders/lags (see Multimedia Appendix 1 for detailed information about the AR model). The ARIMA model consists of both AR(*p*) and MA(*q*) as well as an order *d* differencing term, resulting in the following ARIMA (*p*, *d*, *q*) model [23, 24]:



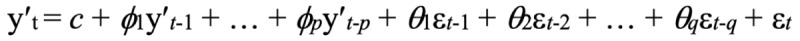



or in backward shift operator form:



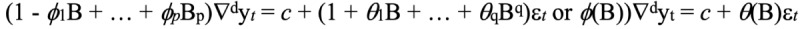



See Multimedia Appendix 1 for details on the parameters.

The ARIMAX model further extends ARIMA to the multivariate time series by incorporating at least one exogenous independent variable *x_t_*. ARIMAX (*p*, *d*, *q*) is specified as follows [25]:



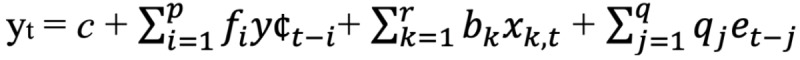



or in backward shift operator form [26]:



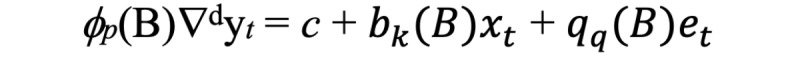



where 
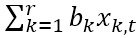
 contributes to the exogeneous independent variable that could potentially influence the dependent variable *y_t_*.

In this study, ARIMAX was developed to evaluate how real-world epidemic metrics, modeled as exogeneous variables, impact CDC tweet topic dynamics as dependent variables. Each of the 4 CDC tweet topics was modeled as a dependent variable (y_t_) and each of the 4 COVID-19 epidemic measures was an independent exogeneous variable (x_t_). The optimal ARIMA and ARIMAX model parameter set (*p, d, q*) was determined by the *R* ARIMA model package.

In addition to reporting the values of the ARIMAX model parameter set (*p*, *d*, *q*), difference in Akaike information criterion (dAIC), root mean square error (RMSE), and mean absolute error (MAE) were also computed to compare different ARIMAX performances. The optimal model was the one with the lowest AIC score. dAIC was computed in between 2 models (see Multimedia Appendix 1 for detailed information on AIC). We had an ARIMA model of a single topic time series and an ARIMAX model of that topic time series with an exogeneous variable. Negative dAIC values indicated that the ARIMAX model showed improvement in model performance over the ARIMA counterpart that did not include an exogenous variable.

The commonly used RMSE and MAE were adopted as performance metrics. They are defined as follows [27]:



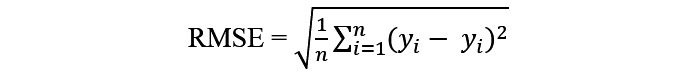





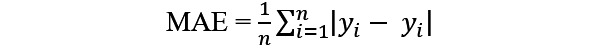



where *n* is the number of data points in a sample y (y*_i_*, where *i*=1, 2, …, *n*). RMSE and MAE are Euclidean distance and Manhattan distance in high-dimensional space, respectively.

## Results

### Topic Modeling and Content Results

A total of 17,524 English tweets posted by the CDC were retrieved and analyzed. Four key topics were generated via LDA based on evaluation metrics including perplexity and coherence score. These topics were then examined and categorized to themes by domain experts (Textbox 1 with example tweets with their respective topics). The themes of the topics and their top 10 unique associated keywords are presented in Table 3.

Topics were plotted as circles and displayed on the left panel; the most relevant terms or associated keywords with their corresponding topics were displayed in frequency bars on the right panel, which showed each term’s frequency from each topic across the corpus (ie, all CDC COVID-19 tweets sampled) [28] (see Multimedia Appendix 1 for more detailed information about visualizations in the pyLDAvis package). The size of the circle indicated the prevalence of that topic in the corpus. Visualizations for all topics, displayed in circles on the left panel, and their top 15 corresponding relevant terms or associated keywords, displayed in frequency bars on the right panel, are provided in Figures S1-S5 in Multimedia Appendix 1.

**Figure 1:**
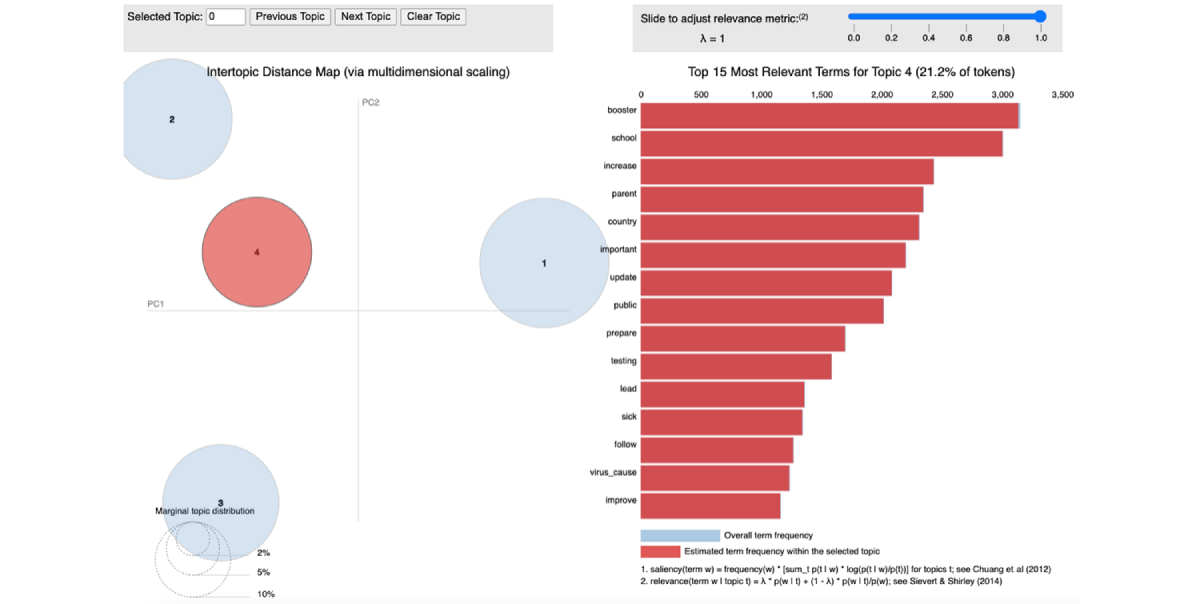
Interactive mapping of topic 4 generated by latent Dirichlet allocation.

Based on the LDA visualization results, these 4 identified key topics had the largest distances and minimal dimensional overlap in the reduced 2D plane. From a public health perspective, the CDC’s online health communication of COVID-19, the largest health emergency in the 21st century, has been relatively cohesive and comprehensive. Therefore, the 4 key topics identified via LDA were not completely mutually exclusive. In addition, the 4-topic model had the balance of separation of topics from a computational perspective and clear interpretability from a health perspective. Increasing the number of topics yields a substantial amount of topic overlap, which was also challenging to provide explicit and clear interpretations. Figure 1 illustrates an example of topic 4 [29,30]. A list of associated terms of topic 4 and the overall frequency of the terms in the corpus have been displayed in the right panel. The 5 key terms from topic 4 based on overall frequency across all tweets were “booster,” “school,” “increase,” “parent,” and “country.”

**Example tweets from each topic theme.**

**Topic 1: General vaccination information and education, especially preventing adverse health outcomes of COVID-19**

- “Even as the world’s attention is focused on #COVID19, this week we are taking time to highlight how #VaccinesWork and to thank the heroes who help develop and deliver lifesaving vaccines. #WorldImmunizationWeek message”
- “CDC’s #COVID19 Vaccine Webinar Series is a great place to start learning about a variety of topics around COVID-19 vaccination.”
- “The #DeltaVariant of the virus that causes #COVID19 is more than two times as contagious as the original strain. Wear a mask indoors in public, even if vaccinated and in an area of substantial or high transmission. Get vaccinated as soon as you can.”

**Topic 2: Pediatric intervention, pediatric vaccination information, family safety, and school and community protection**

- “Make #handwashing a family activity! Explain to children that handwashing can keep them healthy. Be a good role model—if you wash your hands often, your children are more likely to do the same. #COVID19”
- “Parents: During #COVID19, well-child visits are especially important for children under 2. Schedule your child’s routine visit, so the healthcare provider can check your child’s development & provide recommended vaccines.”
- “It is critically important for our public health to open schools this fall. CDC resources will help parents, teachers and administrators make practical, safety-focused decisions as this school year begins.”

**Topic 3: Updates on COVID-19 testing, case, and death data, and relevant information of the disease**

- “CDC tracks 12 different forecasting models of possible #COVID19 deaths in the US. As of May 11, all forecast an increase in deaths in the coming weeks and a cumulative total exceeding 100,000 by June 1. See national & state forecasts.”
- “The latest CDC #COVIDView report shows that the percentage of #COVID19-associated deaths has been on the rise in the United States since October and has now surpassed the highest percentage seen during summer.”
- “#COVID19 cases are going up dramatically. This increase is not due to more testing. As the number of cases rise, so does the percentage of tests coming back positive, which shows that COVID-19 is spreading.”

**Topic 4: Research, study, health care, and community engagement to curb COVID-19**

- “Our Nation’s medical community has been vigilant and their help in identifying confirmed cases of #COVID19 in the United States to date has been critical to containing the spread of this virus.”
- “In a new report using data from Colombia, scientists found that pregnant women with symptomatic #COVID19 were at higher risk of hospitalization & death than nonpregnant women with symptomatic COVID-19. HCPs can inform pregnant women about how to stay safe.”
- “A new study finds masking and fewer encounters or less time close to persons with #COVID19 can limit the spread in university settings. #MaskUp when inside indoor public places regardless of vaccination status.”

**Table 3:** Identified key topics of Centers for Disease Control and Prevention tweets with unique focal keywords.

Key topics	Top 10 unique keywords
1. General vaccination information and education, especially preventing adverse health outcomes of COVID-19 (including cases, severe conditions/hospitalization, and death)	learn, time, safe, fully vaccinate, prevent, child age, old, share, flu, month
2. Pediatric intervention, pediatric vaccination information, family safety, and school and community protection	work, school, datum, test, infection, family, free, home, public, check
3. Updates on COVID-19 testing, case, and death data, and relevant information of the disease	patient, update, booster, cause, recommend, increase, day, program, important, read
4. Research, study, health care, and community engagement to curb COVID-19	vaccination, vaccinate, child, protect, protection, report, visit, risk, community, travel

### Multivariate Time Series Analysis Results

#### CCF Results

The time series of CDC tweet topics and COVID-19 metrics were plotted to visually examine patterns and potential associations. A total of 16 time series plots (4 topics × 4 COVID-19 epidemic metrics) were generated (Figures S14-S29 in Multimedia Appendix 1). CCFs were computed to quantify the dynamic association between each CDC key topic series and each of the 4 COVID-19 epidemic metrics. Quantitative results have been presented (Tables S3-S6 in Multimedia Appendix 1). Visualizations (Figures S30-S44 in Multimedia Appendix 1) illustrated CCFs between both types of time series. CCF values and plots showed that the CDC’s key COVID-19 tweet topic series was not substantially correlated with the confirmed COVID-19 case count series. As an example, there were no specific patterns between topic 2 and daily confirmed COVID-19 cases (Figure 2A).

**Figure 2:**
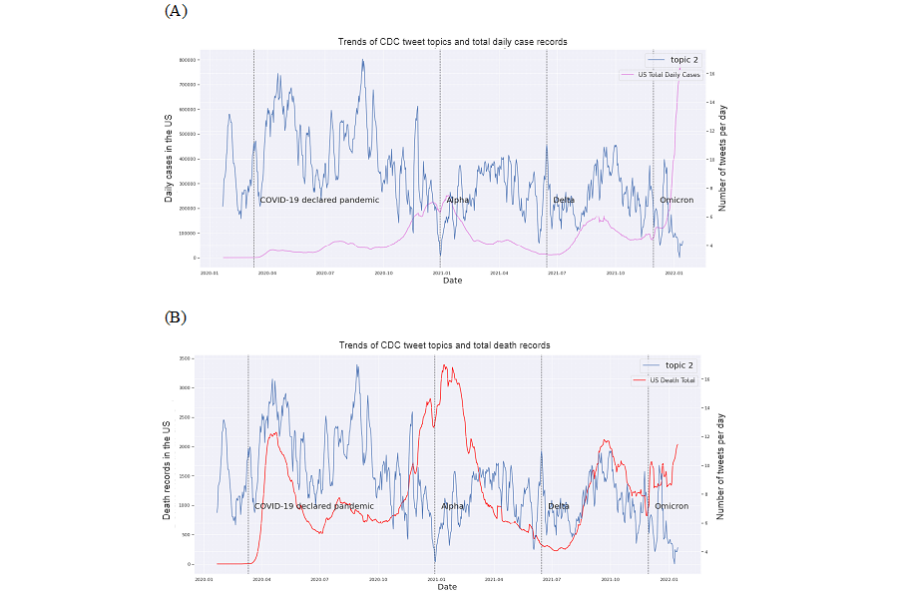
Time series of topic 2 against 2 COVID-19 metrics: (A) case counts, (B) death counts. CDC: Centers for Disease Control and Prevention; US: United States.

**Figure 3:**
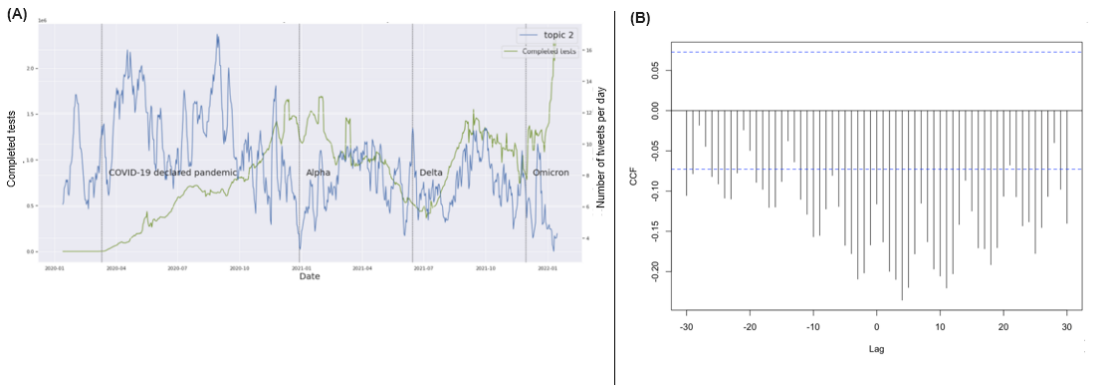
Cross-correlation function (CCF) between the completed COVID-19 test series and topic 2 tweets. (A) Trends of CDC tweet topics and number of completed tests; (B) CCF between COVID-19 confirmed cases and topic 2 tweets. CDC: Centers for Disease Control and Prevention.

**Figure 4:**
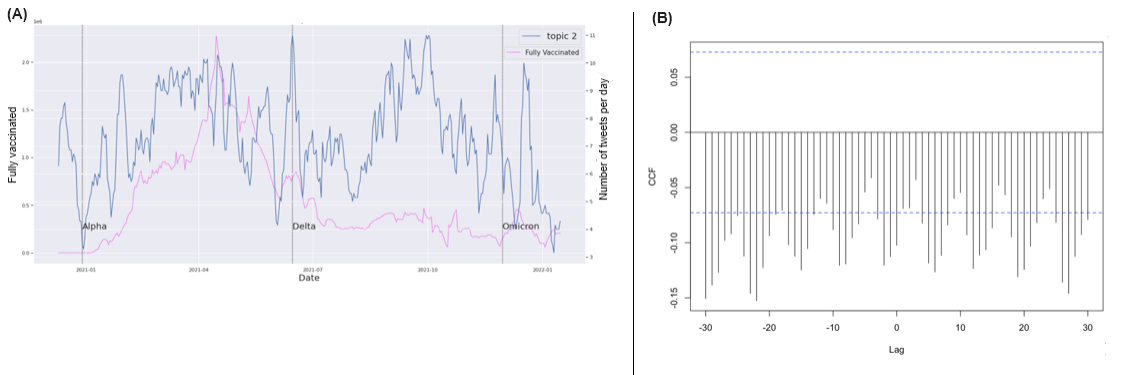
Cross-correlation function (CCF) between the completed COVID-19 vaccination series and topic 2 tweets. (A) Trends of CDC tweet topics and vaccination records; (B) CCF between records of fully vaccinated people and topic 2 tweets. CDC: Centers for Disease Control and Prevention.

COVID-19 confirmed cases and the death time series had very similar dynamic patterns in the United States across the time span (Figure 2B). Consequently, they also showed similar CCFs with the CDC key topic series (Figure S45 in Multimedia Appendix 1). COVID-19 deaths had no substantial correlations with any of the 4 CDC key topics (Figures S18-S21 in Multimedia Appendix 1) based on CCFs. There were no substantial correlations between any of the 4 key topics and the COVID-19 testing series as well as the fully vaccinated rate series. Examples showed the CCFs between those and topic 2 (Figures 3 and 4). These results indicated a potential discrepancy between the CDC’s health communication focus and the actual COVID-19 epidemic dynamics in the United States during the pandemic.

#### MI Results

MI values between each CDC tweet topic and each COVID-19 metric were calculated, and they are shown in Table 4. Confirmed case counts and topic 4 (research, health care, and community engagement to restrain COVID-19) had the highest MI value (3.21), indicating that there was a strong dependency in COVID-19 cases and topic 4. On the other hand, the vaccination rate and topic 3 had the lowest MI value (0.56), indicating almost independence between the 2 series. Among all 4 key topics, topic 4 showed the highest MI values (3.21, 3.02, 3.21, and 1.65) with the 4 COVID-19 metrics. Topic 2 (pediatric intervention, family safety, and school and community protection) had consistently lower MI values with the COVID-19 metric than topic 4. The MI of topic 1 (information on COVID-19 vaccination and education on preventing its adverse health outcomes) and topic 3 (updates on COVID-19 testing, case, and death metrics, and relevant information of the disease) showed similar values with all 4 COVID-19 metrics, although the MI values of topic 1 were slightly higher. Vaccination and educational information on the adverse health outcomes of COVID-19 appeared to not be substantially correlated with COVID-19 epidemic metrics, including testing, cases, and deaths. We speculated that the CDC considered both vaccination and preventing adverse health outcomes of COVID-19 critical to public health and disseminated these topics regardless of the current COVID-19 situation at the time of posting.

In addition, MI values between all pairs of CDC topics were calculated (Table S7 in Multimedia Appendix 1). The resulting MI values, ranked from the largest to smallest, were for topics 2 and 4, topics 3 and 4, topics 1 and 2, topics 2 and 3, topics 1 and 4, and topics 1 and 3. Based on the CDC’s COVID-19 tweeting patterns, pediatric intervention and family and community safety were strongly associated with health care research studies and public engagement to curb the spread of COVID-19.

**Table 4:** Mutual information values between Centers for Disease Control and Prevention key topics and COVID-19 metrics in the United States.

COVID-19 daily measurements in the United States	Topic 1^a^	Topic 2^b^	Topic 3^c^	Topic 4^d^
Confirmed case counts	1.25	2.93	1.18	3.21
Death counts	1.12	2.74	1.06	3.02
Completed COVID-19 test counts	1.24	2.91	1.18	3.21
Fully vaccinated counts	0.60	1.49	0.56	1.65

^a^Topic 1: General vaccination information and education, especially preventing adverse health outcomes of COVID-19.

^b^Topic 2: Pediatric intervention, pediatric vaccination information, family safety, and school and community protection.

^c^Topic 3: Updates on COVID-19 testing, case, and death data, and relevant information of the disease.

^d^Topic 4: Research, study, health care, and community engagement to curb COVID-19.

#### ARIMAX Results

ARIMAX performance measures, including values of ARIMAX parameters (*p*, *d*, *q*), dAIC, RMSE, and MAE, are reported in Table 5. As an external input, the vaccination rate time series significantly improved the performances of the original ARIMA models for all CDC key topics (dAIC = −108.15, −69.79, −90.54, and −91.53 for topics 1 to 4, respectively). This was the largest increase in model performance across all topics with the exogeneous variable in the ARIMAX model. The COVID-19 case series improved the ARIMA model performance for CDC topics 1 and 3 (dAIC = −104.76 and −1.53 for topics 1 and 3, respectively). Including the death or testing series did not result in substantial improvements to the ARIMA model performance for all CDC key topics.

ARIMAX models with lower RMSE and MAE values indicated higher accuracy of the time series models (Table 5). Overall, ARIMAX models for topics 1 and 3 with all COVID-19 metrics delivered the smallest RMSE values (lowest [1.10] for topic 3 with death counts and highest [1.21] for topic 1 with full vaccination records), while those of topic 4 delivered the largest RMSE values (lowest [6.25] with death counts and highest [6.93] with full vaccination records). Similarly, MAE values were the lowest for ARIMAX models for topics 1 and 3 with the epidemic metrics (lowest [0.82] for topic 3 with death counts and highest [0.91] for topic 1 with full vaccination records), and they were the largest for topic 4 with the epidemic metrics (lowest [4.97] with death counts and highest [5.56] with full vaccination records). These ARIMAX performance results showed significant variabilities between the 2 types of time series (CDC key tweet topics and actual COVID-19 metrics in the United States).

We performed an exhaustive search to identify the optimal ARIMAX parameters (*p, d, q*). For example, topic 1 with death counts and completed testing records had the same parameter set (*p*, *d*, *q*=2, 1, 3), indicating that the optimal ARIMAX model between these time series needed first-order differencing (*d*=1) to achieve stationarity and minimal AIC values, its AR time lag was 2 (*p*=2), and its MA time lag was 3 (*q*=3). The topic 1 series with case counts and complete vaccination had the same parameter values (*p*, *d*, *q*=5, 1, 0), indicating that the model was simply an AR model (*q*=0 with no MA terms) with a time lag of 5 (*p*=5) after first-order differencing (*d*=1). The complete ARIMAX parameters are shown in Table 5. All ARIMAX models needed first-order differencing (*d*=1) to be stationary and to minimize AIC values.

**Table 5:** Autoregressive integrated moving average with external variable performance measures of each Centers for Disease Control and Prevention topic and COVID-19 epidemic metric pair.

COVID-19 epidemic measures and ARIMAX^a^ metrics			Topic 1^b^		Topic 2^c^		Topic 3^d^		Topic 4^e^
**Case counts**									
	ARIMAX par^f^	(5, 1, 0)		(4, 1, 1)		(2, 1, 1)		(3, 1, 2)	
	dAIC^g^	−104.76^h^ (2240.19, 2344.95)^i^		0.45 (4304.09, 4303.64)		−1.53^h^ (2227.59, 2229.12)		11.97 (4785.89, 4773.92)	
	RMSE^j^	1.21		4.66		1.12		6.45	
	MAE^k^	0.90		3.66		0.86		5.10	
**Death counts**									
	ARIMAX par	(2, 1, 3)		(4, 1, 1)		(2, 1, 1)		(3, 1, 2)	
	dAIC	6.72 (2240.19, 2233.47)		36.60 (4304.09, 4267.49)		20.43 (2227.59, 2207.16)		60.14 (4785.89, 4725.75)	
	RMSE	1.12		4.56		1.10		6.25	
	MAE	0.84		3.57		0.82		4.97	
**Testing**									
	ARIMAX par	(2, 1, 3)		(4, 1, 1)		(0, 1, 2)		(3, 1, 2)	
	dAIC	0.13 (2240.19, 2240.06)		19.56 (4304.09, 4284.53)		1.83 (2227.59, 2225.76)		36.97 (4785.89, 4748.92)	
	RMSE	1.13		4.60		1.11		6.34	
	MAE	0.84		3.61		0.85		4.99	
**Vaccination**									
	ARIMAX par	(5, 1, 0)		(5, 1, 0)		(5, 1, 0)		(5, 1, 0)	
	dAIC	−108.15^h^ (2240.19, 2348.34)		−69.79^h^ (4304.09, 4373.88)		−90.54^h^ (2227.59, 2318.13)		−91.53^h^ (4785.89, 4877.42)	
	RMSE	1.21		4.90		1.18		6.93	
	MAE	0.91		3.81		0.89		5.56	

^a^ARIMAX: autoregressive integrated moving average with external variable.

^b^Topic 1: General vaccination information and education, especially preventing adverse health outcomes of COVID-19.

^c^Topic 2: Pediatric intervention, pediatric vaccination information, family safety, and school and community protection.

^d^Topic 3: Updates on COVID-19 testing, case, and death data, and relevant information of the disease.

^e^Topic 4: Research, study, health care, and community engagement to curb COVID-19.

^f^ARIMAX parameters (p, d, q).

^g^dAIC: delta Akaike information criterion (AIC) or difference in AIC.

^h^Negative dAIC: indicates improvement of performance in the ARIMAX model compared with its autoregressive integrated moving average (ARIMA) model.

^i^AIC values of ARIMA and its corresponding ARIMAX models.

^j^RMSE: root mean square error.

^k^MAE: mean absolute error.

## Discussion

### Principal Findings

In this study, we systematically investigated and comprehensively identified the CDC’s key topics, COVID-19 epidemic metrics, and dynamic associations between the 2 types of data series based on 17,524 COVID-related English tweets from the CDC since January 2022. The LDA topic model was built to characterize and identify the dynamic shifts of topics in the CDC’s COVID-19 communication over a period of more than 2 years. For the first time, we were able to identify the following 4 key topics: (1) general vaccination information and education; (2) pediatric intervention that also involved family and school safety; (3) updates on the COVID-19 epidemic situation, such as numbers of cases, deaths, etc; and (4) research studies that were able to curb the pandemic.

Our study took a unique approach of infoveillance by identifying potential associations between COVID-19 epidemic outcome metrics in the United States and the CDC’s key topic dynamics during different stages of the pandemic. This innovative framework significantly expanded the original infoveillance approach that generally relied on the number of posts (ie, posting dynamics) without further extracting more detailed and meaningful content topics and sentiments from the textual data. Our study was able to further provide practical and useful health communication strategies for public health agencies to effectively communicate timely and accurate information to the public. It is important to investigate the dynamic associations between the CDC’s tweets on COVID-19 and the progression of the pandemic for several reasons:

1. Understanding their relationship can reveal how public health messaging impacts public perception and engagement at different stages of a major health emergency. A strong association between the CDC’s tweets and epidemic measures indicates that public health messaging is effective. Weak associations might indicate that messaging from the CDC to the public over time is not effective; however, it will lead us to further explore the influential factors and provide health communication strategies for public health agencies.
2. It can also show if the CDC’s messaging on Twitter is proactive or reactive to the actual epidemic, informing strategies for future public health communication.
3. It helps public health agencies better allocate resources. For example, if tweets related to educating the public on monitoring COVID-19 symptoms and updating certain metrics lead to an increase in the number of people trying to get COVID tests, then resources could be directed toward opening testing centers and sending free test kits to homes.

**Figure 5:**
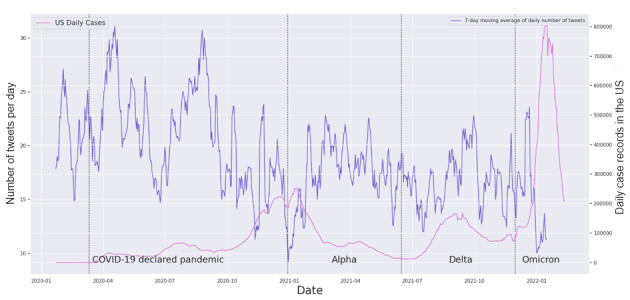
Time series of the daily number of Centers for Disease Control and Prevention (CDC) tweets and COVID-19 case counts. US: United States.

Our study is the first of its kind to comprehensively evaluate the impact of online public health communication, especially on Twitter, which is one of the major social media platforms, during different phases of a large health emergency. We studied the overall daily volume of COVID-19–related tweets posted by the CDC over time as a baseline (Figure 5), and the volume of tweets was higher in the early phase of the pandemic, indicating a strong effort at the CDC to disseminate important information to the public. We did not observe visually clear patterns of an association with COVID-19 epidemic measures. We further applied novel NLP to significantly reduce the gap of previous studies that overlooked the dynamic association between detailed topics discussed by public health agencies on social media and real-world epidemic metrics.

We then examined the dynamic associations between the 4 identified key topics and 4 COVID-19 epidemic outcome metrics. Among the 4 major topics, topic 1, which covered information on vaccination and adverse health outcomes of COVID-19, had substantially strong associations with death counts and testing records during the Alpha phase (December 29, 2020, to June 14, 2021). We found that during this phase, when the overall vaccination-related CDC tweets were decreasing, the daily vaccination rate (number of people who received a complete primary series of the COVID-19 vaccine based on the CDC Vaccine Tracker) was increasing, which aligned with the CDC’s effort in emphasizing the importance of vaccination to the public on social media. When discussions from the CDC about vaccination were increasing after the Alpha phase, the vaccination rate started to decrease. The reasons could be but are not limited to the following:

1. Ineffective messaging from the CDC on social media to the public during later stages of the pandemic.
2. Lack of engagement from the public, since not everyone follows or engages with official accounts and might miss or overlook them amidst other content.
3. Fatigue from information overload where frequent data updates on social media platforms can lead to desensitization, making it less likely for users to pay attention over time and act on the information.
4. Temporal delays create time lag, which can impact the associations between the topics and the real epidemic measures.
5. Political factors such as antivaccination groups.

Therefore, with all possible influential factors, the CDC could not fully impact the public’s responses and actions on getting vaccinated even though they had been making efforts on sharing educational information about vaccination. This finding showed that the CDC had been making efforts to emphasize the importance of vaccination on Twitter, but the public response was weak. Thus, it is important to further study the influential factors for the CDC’s social media strategies. Topic 3, which provided updates on 3 of the COVID-19 measures (testing, cases, and deaths) and their relevant information, aligned better with the case series during the Delta phase (June 15, 2021, to November 29, 2021). It also matched with the death series during the declared pandemic phase (original variant: March 11, 2020, to December 28, 2020) and Delta phase, classified by the World Health Organization on May 11, 2021. Furthermore, even though topic 3 did not demonstrate a visible association with the testing series, timely communication from the CDC was actually strongly associated with the testing time series over the entire study period based on the multivariate time series analysis.

According to these key findings, we suggest that aligning the content topics of health communication from public health agencies with the temporal dynamics of COVID-19 or other emerging public health emergencies (eg, major epidemic outcome metrics) can help provide more timely and relevant information to the public. Therefore, we recommend that the CDC and other public health agencies monitor the epidemic outcome metrics in real time. Health agencies can then post timely updates about the emergency, most recent findings, and interventions on social media according to the dynamic changes of these outcome metrics. Public health agencies can regain trust from the public by not only helping the public better understand the complex dynamics of the health emergency, but also informing the public with evidence-based guidance and recommendations more effectively.

### Limitations and Future Work

There are several limitations in this infoveillance study that could be improved in future work. First, while we focused on probabilistic-based LDA for topic modeling, there are other alternative NLP approaches such as deep learning–based bidirectional encoder representations from transformers (BERT). Hence, we will explore BERT and other state-of-the-art NLP techniques for content topic modeling and sentiment analysis in the future. Second, given the complexity of this study, we will incorporate subthemes to further help contextualize the clusters in future work. Third, the CDC does not have the sole power of controlling people’s responses and actions over time (eg, getting tested and receiving full vaccine doses), even with consistent effort on Twitter to educate the public and mitigate the pandemic. There are other factors that could affect the associations between the CDC’s messages and the COVID-19 measures:

1. Time lags: What is posted might not reflect real-time situations, which can impact the association strength between the posted measures and real-world metrics; thus, we suggest aligning the content topics of health communication with up-to-date epidemic outcome metrics.
2. Discrepancies in posting methods: The CDC simplifies the data in their posts to make the information more comprehensible for the audience, which might not align with the detailed epidemic metrics posted from other sources with different interpretations of the reported metrics.
3. Variability in the data source: The data open to the public might come from sources and reporting standards that are different from the CDC’s protocol, which could weaken potential associations.
4. Audience: As a government health agency, the CDC prioritizes certain data for social media to cater to the public for relevancy. For example, posting daily epidemic measures could lead to strong associations with COVID-19 metrics, but an association does not mean causality, and we assume that the CDC does not generate their tweets with the intention to improve associations of any kind and their priority is to present a variety of reliable information to the public.
5. Fatigue from information overload: Frequent data updates on social media can lead to desensitization, making it less likely for users to pay attention and react to the information over time, for example, tweeting about daily epidemic measures decreases the public’s attention over time.
6. Political and societal factors, for example, antivaccination groups and conspiracy theories about the pandemic.

In addition, it is important for us to continue to examine the validity of the underlying assumption that the CDC’s health communication makes an impact during a pandemic. In this infodemiology study, we focused on the national effects of these tweets. Future studies should further examine geospatial factors and other confounding factors to help understand whether and how much the CDC’s tweets impact pandemic outcomes.

Lastly, public engagement (ie, retweets, likes, replies, etc) of the CDC’s health communication is an important indicator of the effectiveness of online health communication efforts. There have been studies that analyzed public sentiments and attitudes [31-34] toward various health-related topics. However, very few studies have investigated the associations of public sentiment shifts along disease-related metrics. In addition, public sentiments and attitudes are heavily influenced by health agencies’ messages and should not be misled by misinformation. Public opinions also influence health practices and interventions, which have a significant impact on the actual epidemic outcomes (eg, case, death, vaccination, etc). Thus, it is important to further investigate the underlying association between public health communication topics and actual epidemic measures. The insights can help public health agencies develop better social media strategies to address public concerns at different stages of the emergency. Therefore, we suggest that examining the dynamics and patterns of public responses to health agencies’ original communications can provide valuable insights on public perceptions and attitudes around various issues during the pandemic, such as pharmaceutical interventions (eg, vaccination) and nonpharmaceutical interventions. Detailed content analysis can be applied to explicitly identify public concerns in response to the CDC’s health communications. In addition, sentiment analysis can be applied to extract public sentiments (ie, positive, neutral, or negative) toward the CDC’s health communications, and further help identify public attitudes and reactions to various content topics that the CDC has communicated. Public attitudes will ultimately determine individual health behavior and decision-making, such as vaccination acceptance and compliance with nonpharmaceutical interventions, which in turn drive the overall epidemic dynamics. Therefore, it is critical to investigate real-time public engagement, such as retweeting or replying on social media, toward public health agencies’ communications to better inform health agencies about prioritizing their communications and addressing public concerns about specific content topics.

### Conclusions

This study investigated the dynamic associations between the CDC’s detailed COVID-19 communication topics on Twitter and epidemic metrics in the United States for almost 2 years during the pandemic. Using LDA topic modeling, we were the first to comprehensively identify and explore various COVID-related topics tweeted by the federal public health agency during the pandemic. We also collected daily COVID-19 epidemic metrics (confirmed case counts, death counts, completed tests records, and fully vaccinated records) and performed various multivariate time series analyses to unravel the temporal patterns and associations with the CDC’s COVID-19 communication patterns (ie, investigated the dynamic associations between the time series of each topic generated by the LDA model and the time series of each epidemic metric). The results suggested that some topics were strongly associated with certain COVID-19 epidemic metrics, indicating that advanced social media analytics (eg, NLP) could be a valuable tool for effective infoveillance. Based on our findings, we recommend that the CDC, along with other public health agencies, could further optimize their health communications on social media platforms by posting contents and topics that align with the temporal dynamics of key epidemic metrics. While the CDC had been making efforts to share information on social media platforms to educate the public throughout the pandemic, the public responses to these messages were relatively weak. It is important to further explore the potential factors that played a role in the effectiveness of the CDC’s social media performance in future studies. As such, we suggest increasing online health communication on health practices and interventions during high-level epidemic periods with large numbers of cases and deaths. Our findings also highlighted the importance of health communication on social media platforms to better respond to and tackle future health emergencies and issues.

## Appendix

Multimedia Appendix 1 Supplementary information.

## Abbreviations

AIC: Akaike information criterion
AR: autoregressive
ARIMA: autoregressive integrated moving average
ARIMAX: autoregressive integrated moving average with external variable
BERT: bidirectional encoder representations from transformers
CCF: cross-correlation function
CDC: Centers for Disease Control and Prevention
dAIC: difference in Akaike information criterion
LDA: latent Dirichlet allocation
MA: moving average
MAE: mean absolute error
MI: mutual information
NLP: natural language processing
PCR: polymerase chain reaction
RMSE: root mean square error
TF-IDF: term frequency-inverse document frequency
