# Supplementary material for: Dynamic Associations Between Centers for Disease Control and Prevention Social Media Contents and Epidemic Measures During COVID-19: Infoveillance Study
Source: JMIR Infodemiology. 2024 Jan 23;4:e49756. doi: 10.2196/49756 (PMC10848128; doi:10.2196/49756)
Supplement: Multimedia Appendix 1 [file infodemiology_v4i1e49756_app1.pdf]

Appendix

Latent Dirichlet allocation (LDA) topic model:

- A corpus is denoted by D = {$\mathbf{w_1}$, $\mathbf{w_2}$, …, $\mathbf{w_M}$}, where M = the number of documents within the corpus
- Each topic for a document is denoted by T = {t_1, t_2, …, t_K}, where K = the number of topics in the corpus
- Each document is denoted by **w** = (w_1, w_2, …, w_N), where N = the number of words in the document
- Each word in the document is indexed by {1,…,V}

LDA algorithm achieves the following tasks:

1. Forming an M x K matrix, where the weight $w_{m,k}$ = the association between a document d_m and a topic t_k.
2. Forming an N x K matrix, where the weight $w_{n,k}$ = the association between a word w_n and topic t_k.

Notations:
Dirichlet(i): Dirichlet distribution with parameter i;
Mult(i): multinomial distribution with parameter i;
β_t: word distribution for topic *t*;
θ_d: topic proportion for document *d*;
η and α: hyperparameters of each of their Dirichlet distributions.
The respective generative processes for each task are shown below [1]:

1. For each topic t ∈ {1, …, K}, draw a distribution over words $\beta_t \sim$ Dirichlet (η): from each document randomly draw words and assign them to K topics.
2. For each document d,
   a. Draw a vector of topic proportions $\theta_d \sim$ Dirichlet (α):
   b. For each word w_n in document d, where n ∈ {1, …, N}
      i. Draw a topic assignment $\mathbf{z}_n \sim \text{Mult}(\theta_d)$: calculate the probability of assignments to topic *t* over all documents that come from word *w*, obtains the number of documents there are in topics *t* from the word w.
      ii. Draw a word $w_n \sim \text{Mult}(\beta_{zn})$: calculate the probability of words in documents *d* that are assigned to topic t, obtains the number of words belong to the topic *t* given document *d*
      iii. The probability of the word *w* that belongs to topic *t* is the product of the above two probabilities

Autoregressive (AR) model:
AR is a regression model where linear combinations of the past terms are the predictors of the variable in the future. Assuming the values of the variable are independently and normally distributed, AR(*p*) model of order or lag *p* can be written as:
$y_t = c + \phi_1 y_{t-1} + \phi_2 y_{t-2} + \ldots + \phi_p y_{t-p} + \varepsilon_t = c + \sum_{i=1}^{p} \phi_i y_{t-i} + \varepsilon_t$,
or in a simplified version with backward shift operator form:
$\phi_p(B)y_t = 1 - \phi_1 B + \ldots + \phi_p B_p$,

where c = average of changes between the sequential observations,
ε_t = white noise,
y_t = lagged values, or predictor values of the next value of the variable y.
MA is another linear model of past forecast errors in a univariate time series. MA(q) model of order or lag q can be written as follows:
$y_t = c + \theta_1\varepsilon_{t-1} + \theta_2\varepsilon_{t-2} + \ldots + \theta_q\varepsilon_{t-q} + \varepsilon_t = c + \sum_{j=1}^{q} \theta_j y_{t-j} + \varepsilon_t$,
or in backward shift operator form:
$\theta_q(B)y_t = 1 - \theta_I B + \ldots + \theta_q B_q$.

Autoregressive integrated moving average (ARIMA) model:
ARIMA model consists of both AR(p) and MA(q) as well as order d differencing term, resulting in the following ARIMA (p, d, q) model:
$y'_t = c + \phi_1 y'_{t-1} + \ldots + \phi_p y'_{t-p} + \theta_1\varepsilon_{t-1} + \theta_2\varepsilon_{t-2} + \ldots + \theta_q\varepsilon_{t-q} + \varepsilon_t$, or in backward shift form:
$(1 - \phi_1 B + \ldots + \phi_p B_p)\nabla^d y_t = c + (1 + \theta_1 B + \ldots + \theta_q B^q)\varepsilon_t$ or $\phi(B)\,)\nabla^d y_t = c + \theta(B)\varepsilon_t$,
where y'_t = time series after differencing, which is presented as $(1 - B)^d y_t$ as d differences in backward shift operator form,
$\nabla^d = (1 - B)^d y_t$,
p = order of past lagged values of for each time point of autoregressive model,
d = the degree of differencing occurred, or the number of times performs integration,
q = order past lagged errors for the error term as a combination of predictors of moving average model.

Akaike information criterion (AIC):
Given a set of data, Akaike information criterion (AIC), which was proposed by Akaike in his published paper in 1974, is used as an estimator for statistical model comparison and selection. It is defined as:

$$\mathrm{AIC} = -2\log(L) + 2k,$$

where k = the number of independent parameters, L = the maximum likelihood for a statistical model. AIC is widely used to estimate the model's fit, especially for time series data, greater penalties are provided accordingly to improve the fit, but could meet its limitation when data size becomes large, leading overfitting of the model. We have performed series of exhaustive searches were executed amongst all possible ARIMA models in this study for model comparison and selection.

Definition of a complete series of vaccine: either one dose of a single-dose vaccine (Johnson & Johnson viral vector vaccine) or two-dose vaccines (Pfizer or Moderna mRNA vaccine).

Time span for data collection:
Start date: December 7, 2019. End date: January 15, 2022

Search query used to obtain CDC tweets for data collection:

(ncov OR ncov-19 OR sars OR SARS-COV-2 OR "corona virus" OR pandemic OR pheic OR "wuhan virus" OR "china virus" OR "wuhan pneumonia" OR "wuhan flue" OR kungflue OR covid19 OR covid OR "covid 19" OR coronavirus OR vaccine OR vaccines OR vaccination)

Data collection process of CDC tweets:
Using the Twitter academic API and search query, we retrieved a total of 17,524 English tweets posted by seven official CDC-affiliated Twitter accounts to January 15, 2022. These accounts are: @CDCgov: CDC's official Twitter source for daily credible health and safety updates from Centers for Disease Control & Prevention; @CDCEmergency: CDC Emergency, which tweets ways to for public health preparedness during emergency responses; @CDCDirector: account of the CDC director of the time; @CDCGlobal: CDC Global health, which tweets about how CDC strives to contribute to saving lives, reducing disease, and improving global health around the world; @CDCtravel: CDC Travel health, which voices to help travelers and their clinicians prevent illness and injury during international travel; @DrKhabbazCDC: past Twitter account from former director of CDC emerging infections (NCEZID), Dr. Rima Khabbaz. NCEZID works to protect people from emerging and zoonotic infectious diseases, from anthrax to Zika; @CDCMMWR: MMWR (Morbidity and Mortality Weekly Reports), which is CDC's primary vehicle for scientific publication of timely, authoritative, and useful public health information and recommendations. We consolidated these tweets into a data frame of daily tweet counts of each of the seven CDC-affiliated accounts during the time period. The associated metadata include tweet posting dates, textual data of the tweets, tweet account ID, account types (organization or individual), public engagement metrics (e.g., number of retweets, replies, likes, and quotes), referenced tweet type (retweeted, replied to, and quoted), and referenced tweet IDs.

Stopwords list used in LDA topic model:
{'covid','continue','good', 'include','mmwr','feel','etc','ever','sure','covid19', 'can','especially', 'many', 'well', 'our','sarscov2', 'ncov19', 'ncovcoronavirus', 'sars', 'pandemic', 'get', 'cdc', 'use', 'see', 'take', 'give', 'thank', 'still', 'show', 'first', 'keep', 'go', 'know', 'also', 'make', 'today', 'find', 'need', 'show', 'vaccine', 'health', 'amp', 'mask', 'outbreak', 'case', 'ppe', 'n95', 'shortage', 'frontline', 'new', 'health', 'help', 'people', 'year'}

The pyLDAvis visualizations are displayed in two left and right panels. Visualization features are displayed on left panel, where topics are presented, and right panels, where the associated keywords with their corresponding topics are displayed in frequency bars:
1. Circles of topics generated by the LDA topic model: displayed on the left panel in different sizes, "intertopic distance map", showing how far or near they are from each other color-coded in blue when not selected. The color of the circle is blue when no topic is being selected and is red when selected. The size indicates the prevalence of that topic in the corpus.
2. List of associated keywords of the topics in the corpus
3. Saliency (blue bars): the overall frequency of a word in the corpus, shown when no word is being selected.
4. Relevance (red bars): shows how much information a word explains about a topic by measuring the frequency of a word within a topic with weight λ.

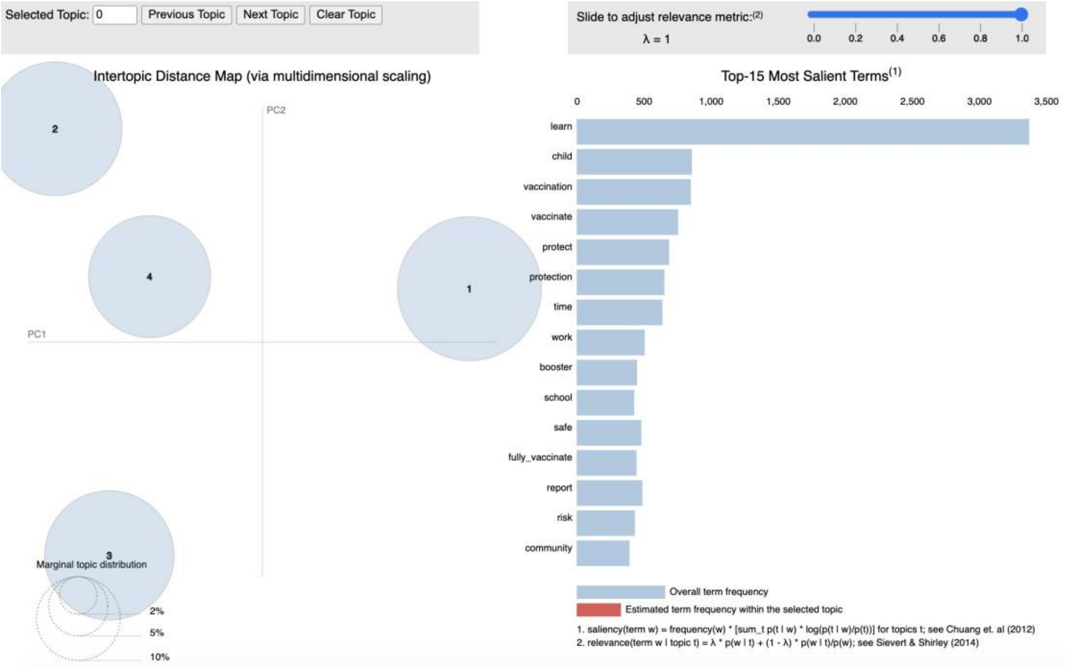

Figure 1. Interactive mapping of the topics generated by LDA

Topic 1 and its associated keywords/relevant terms:

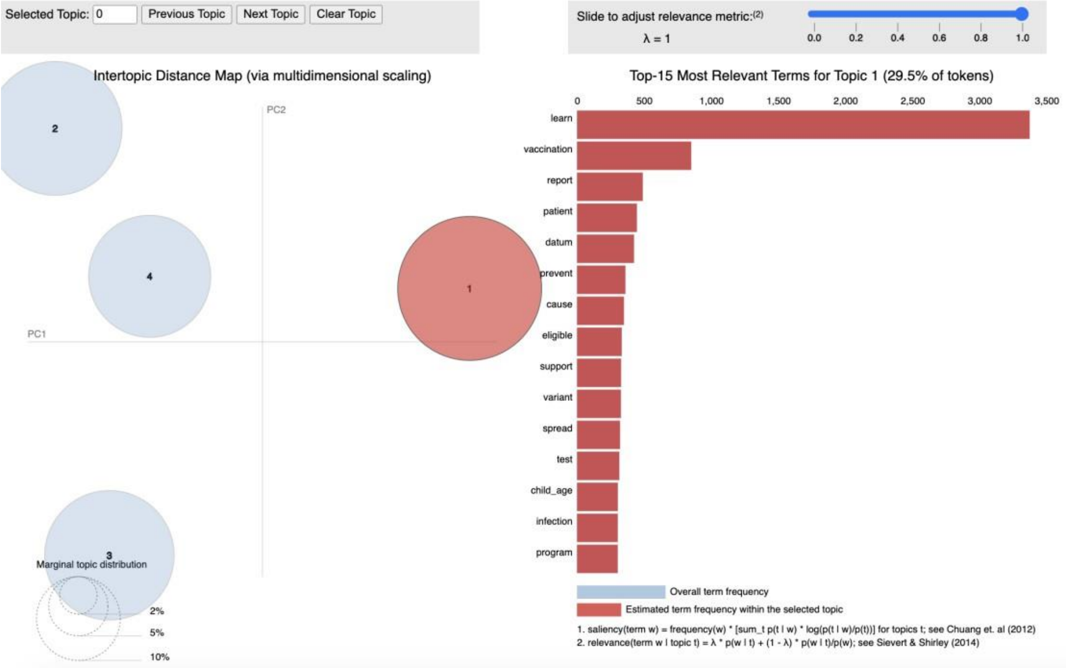

Figure 2. Interactive mapping of topic 1 generated by LDA

Topic 2 and its associated keywords/relevant terms:

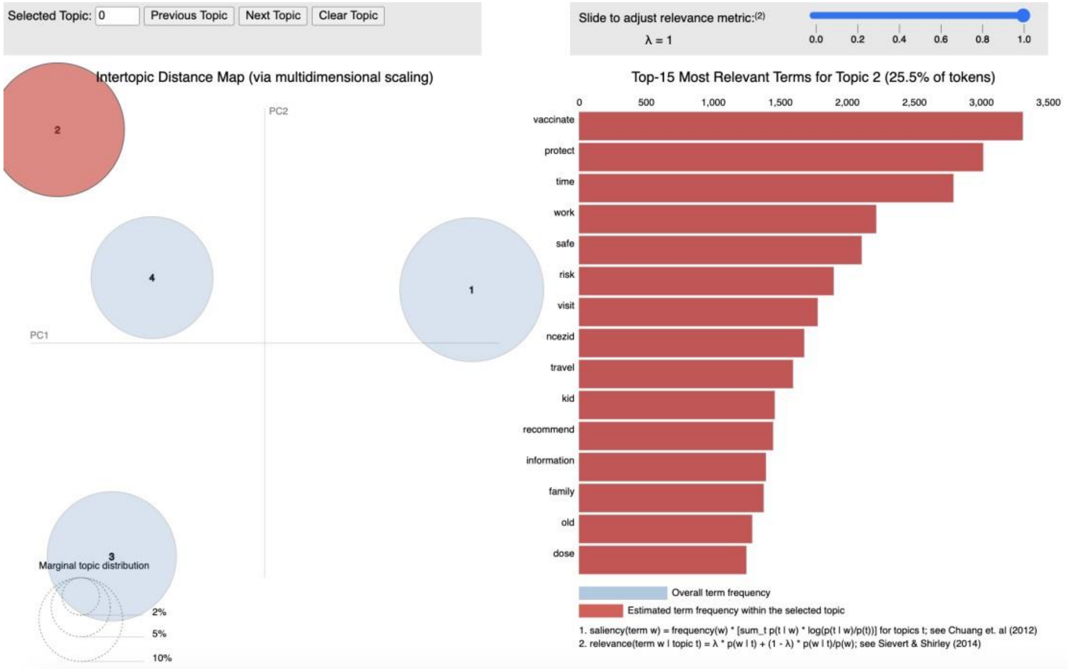

Figure 3. Interactive mapping of topic 2 generated by LDA

Topic 3 and its associated keywords/relevant terms:

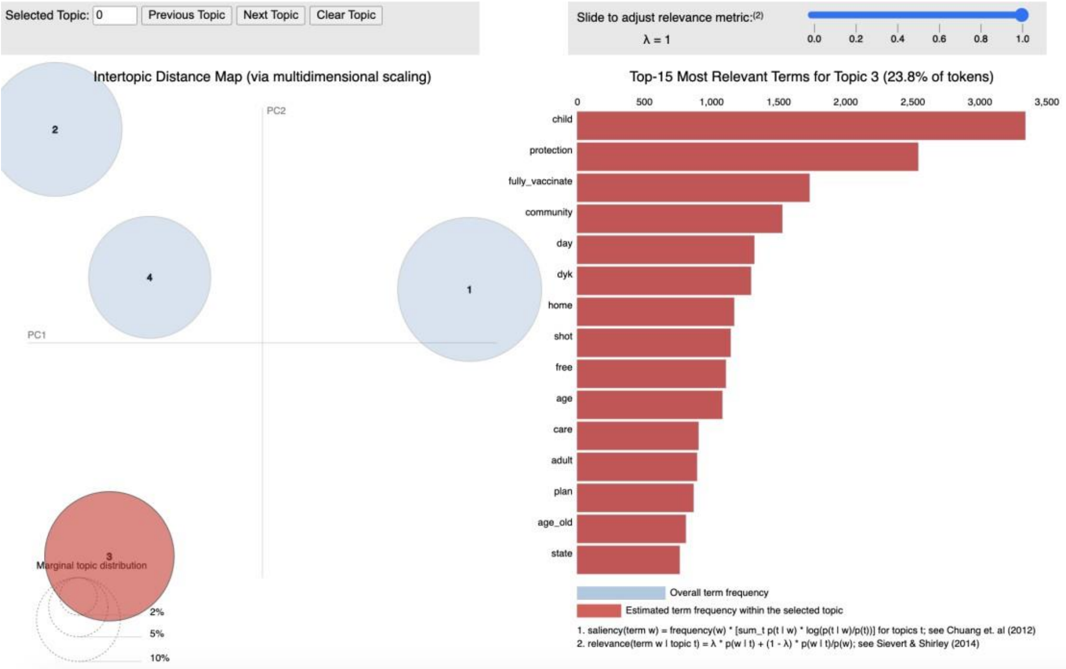

Figure 4. Interactive mapping of topic 3 generated by LDA

Topic 4 and its associated keywords/relevant terms:

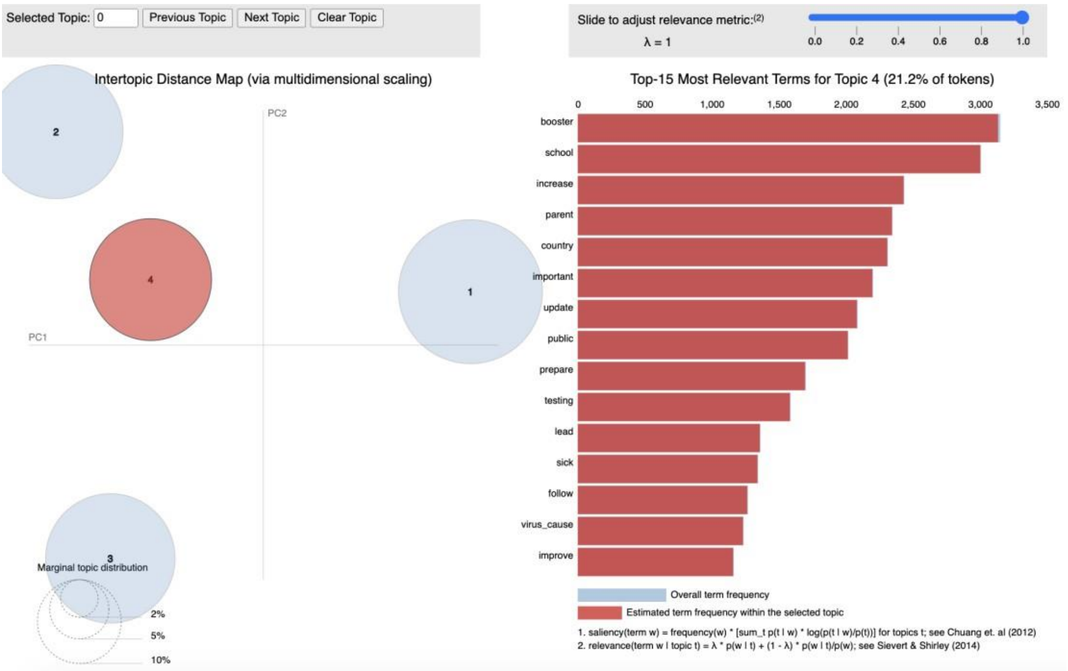

Figure 5. Interactive mapping of topic 4 generated by LDA

Table 1. Identified prominent topics of Centers for Disease Control and Prevention tweets and their respective unique focal keywords.

Key topics	Top 10 unique keywords
1. General vaccination information and education, especially preventing adverse health outcomes of COVID-19 (including cases, severe conditions/hospitalization, and death)	learn, time, safe, fully vaccinate, prevent, child age, old, share, flu, month
2. Pediatric intervention, pediatric vaccination information, family safety, school and community protection	work, school, datum, test, infection, family, free, home, public, check
3. Updates on COVID-19 testing, cases, and deaths data, and relevant information of the disease	patient, update, booster, cause, recommend, increase, day, program, important, read
4. Research, study, healthcare, and community engagement to curb COVID-19	vaccination, vaccinate, child, protect, protection, report, visit, risk, community, travel

Individual time series plots: the four COVID-19 epidemic measurements and the four CDC topics after detrending and testing for stationarity.

X-axis shows the number of days, and y-axis shows the number of the measurement on daily basis (in blue), 7-day moving average (in red), and standard deviations (in green):

- Confirmed cases:

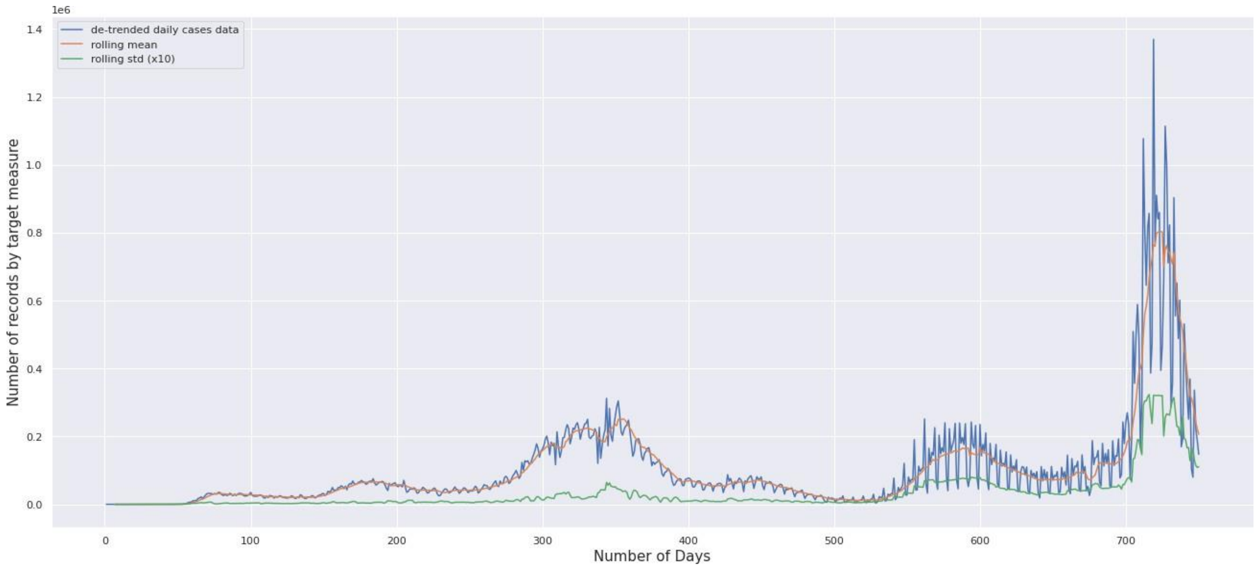

Figure 6.

- Deaths:

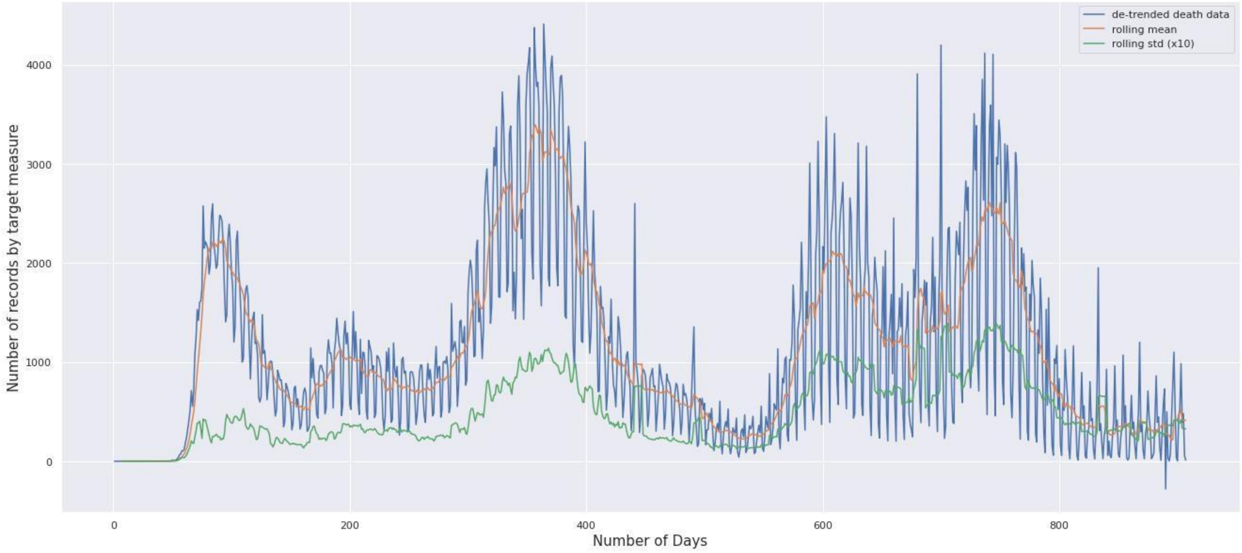

Figure 7.

- Testing:

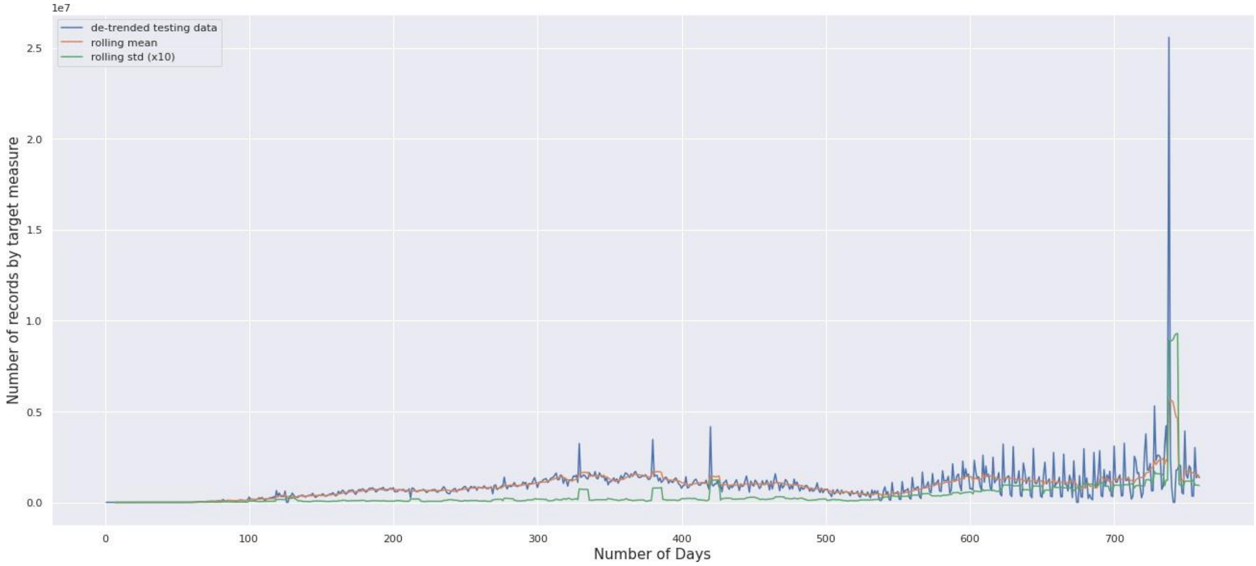

Figure 8.

- Vaccination:

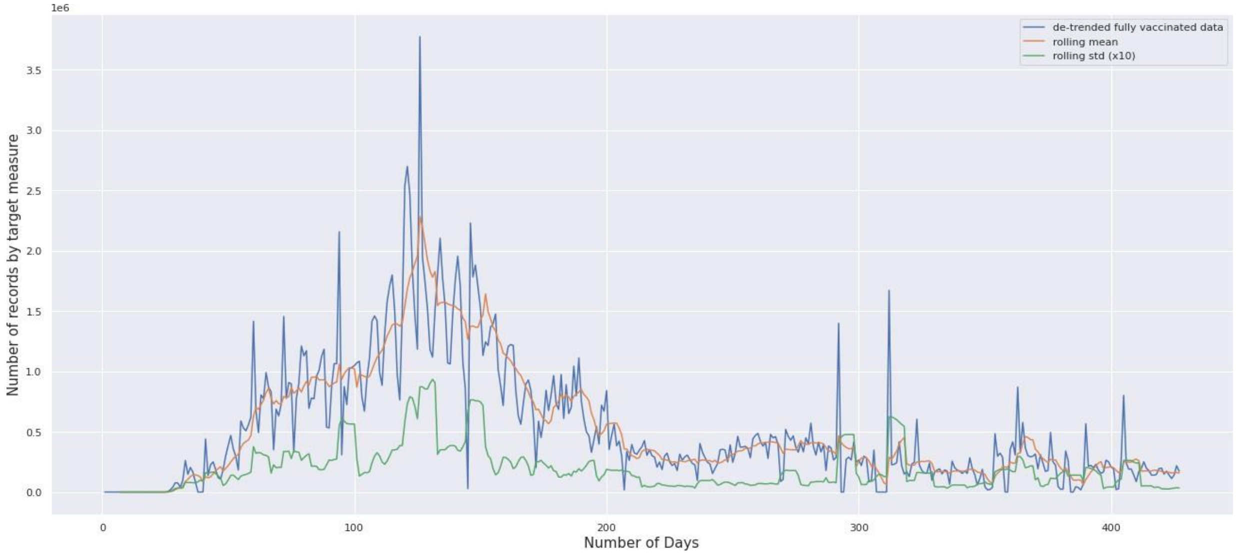

Figure 9.

- Topic 1: General vaccination information and education, especially preventing adverse health outcomes of COVID-19 (including cases, severe conditions/hospitalization, and death)

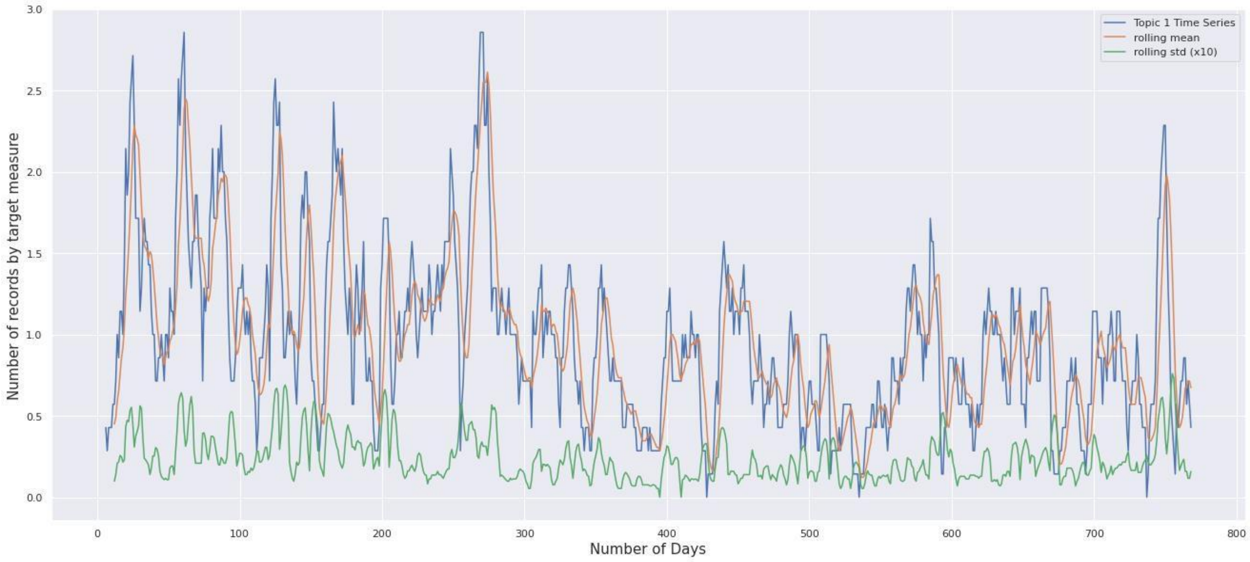

Figure 10.

- Topic 2: Pediatric intervention, pediatric vaccination information, family safety, school and community protection

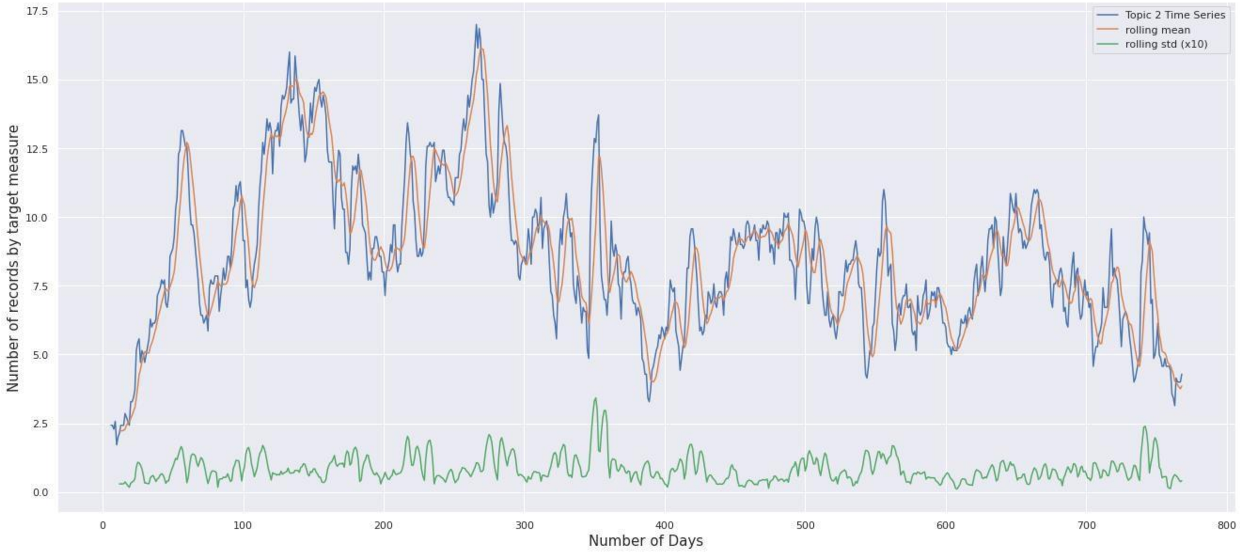

Figure 11.

- Topic 3: Updates on COVID-19 testing, cases, and deaths data, and relevant information of the disease

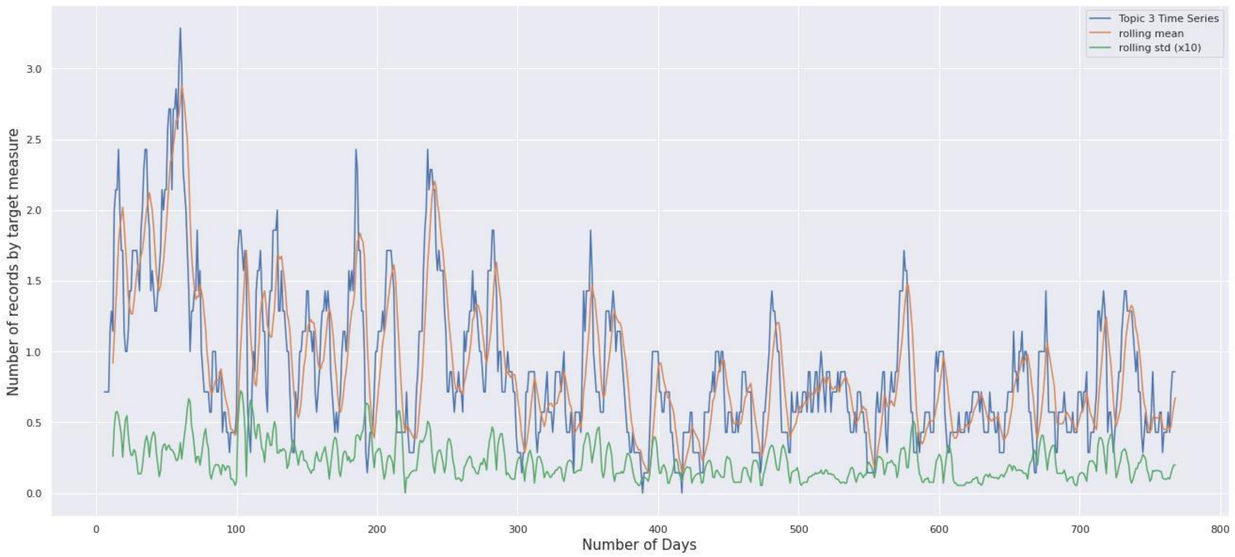

Figure 12.

- Topic 4: Research, study, healthcare, and community engagement to curb COVID-19

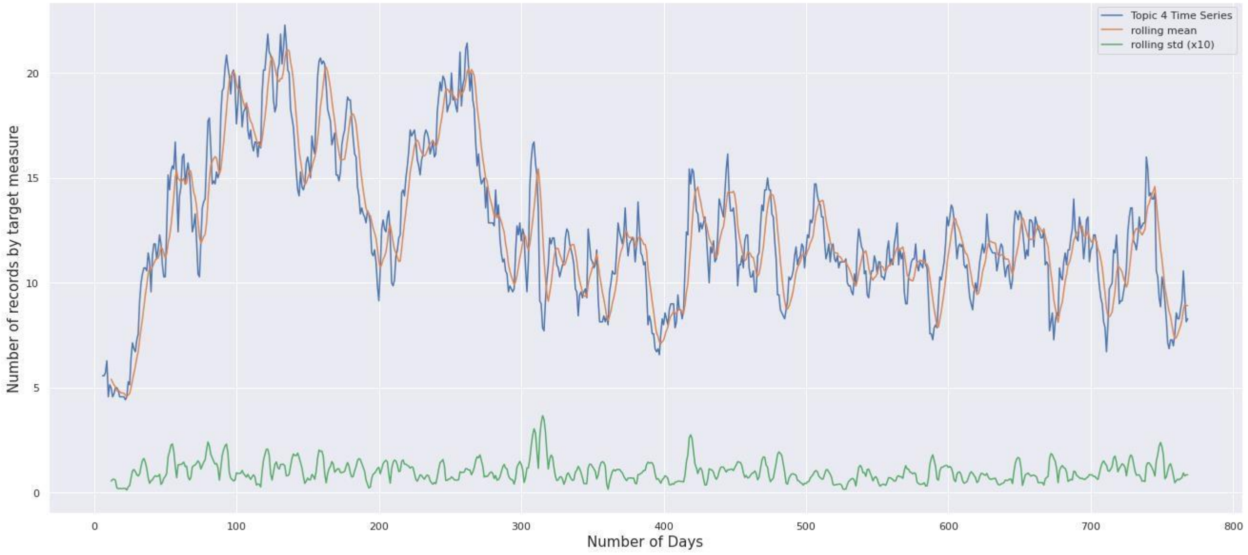

Figure 13.

Time series plots: CDC topics and COVID-19 epidemic measurements in dual axis

- Cases and Topic 1:

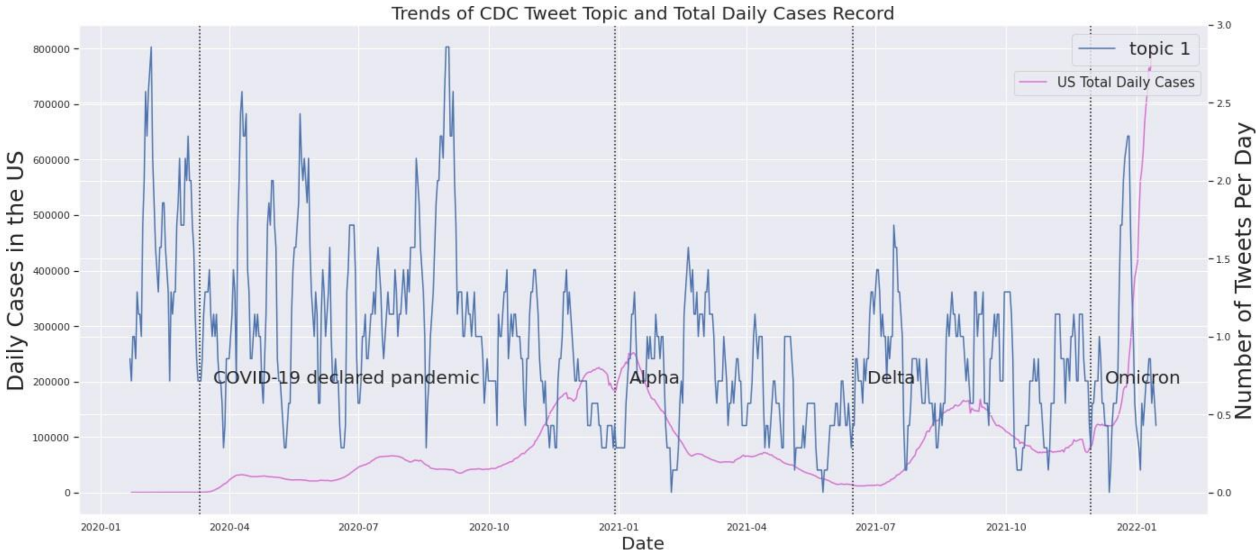

Figure 14.

- Cases and Topic 2:

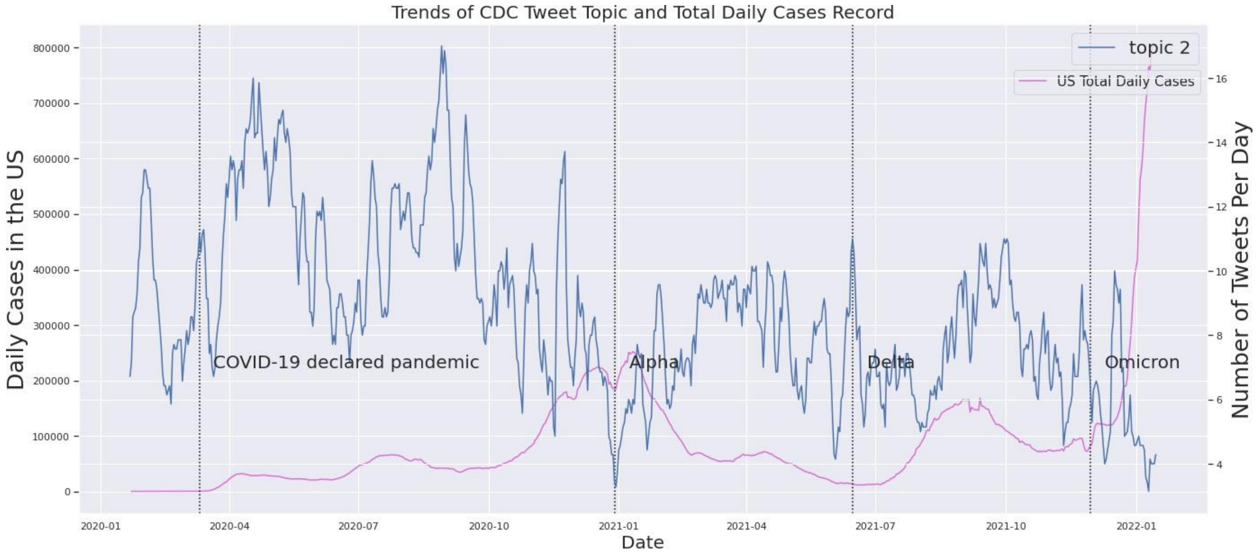

Figure 15.

- Cases and Topic 3:

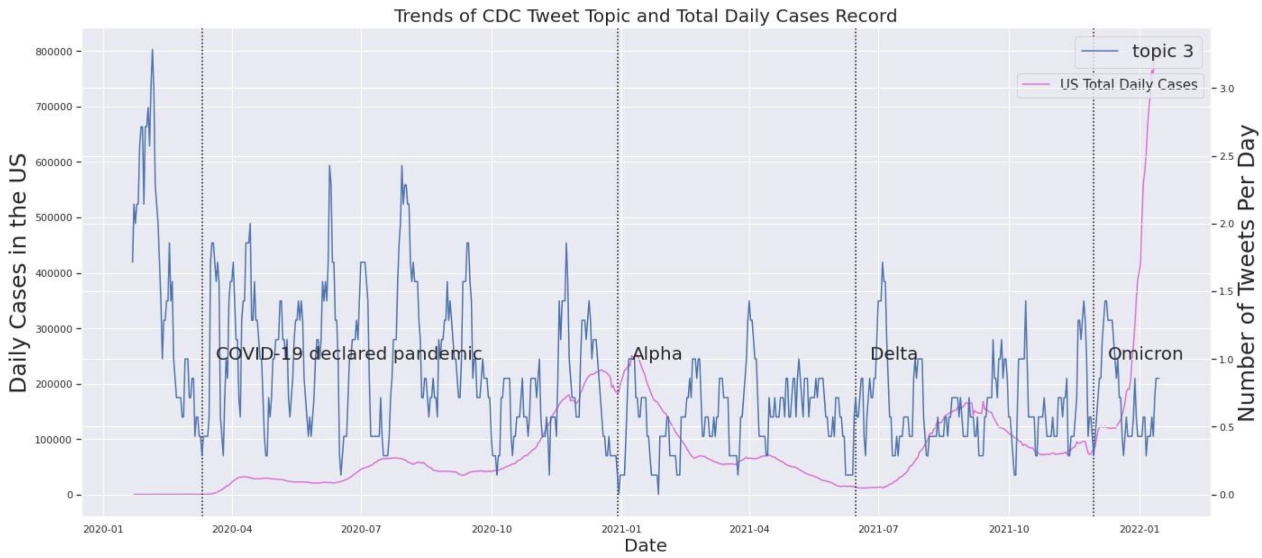

Figure 16.

- Cases and Topic 4:

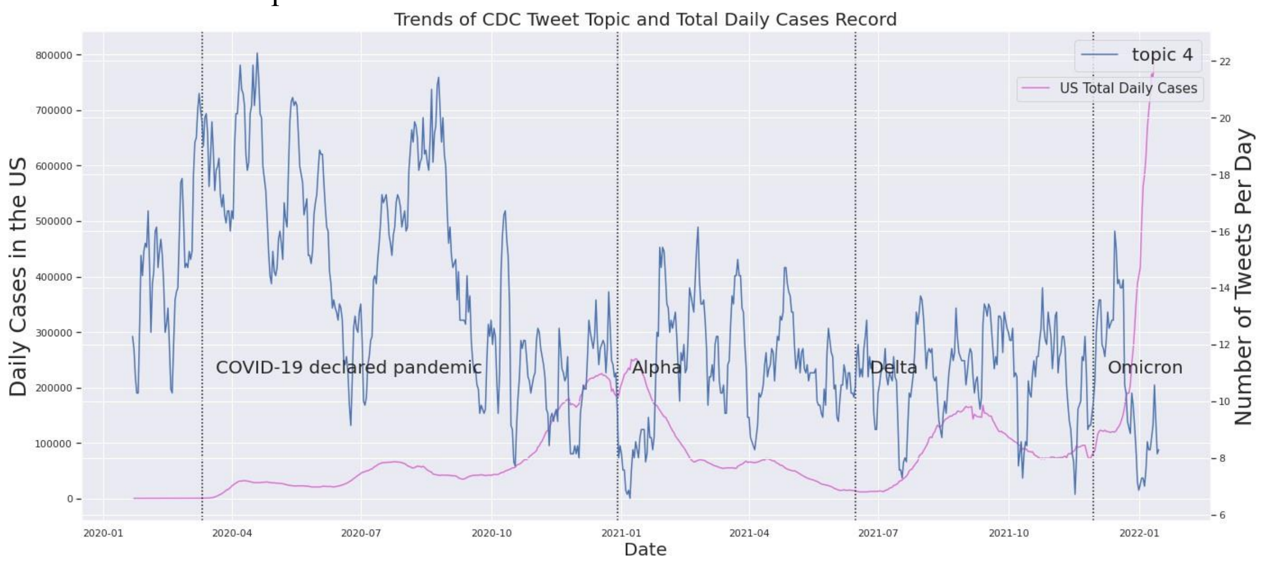

Figure 17.

- Deaths and Topic 1:

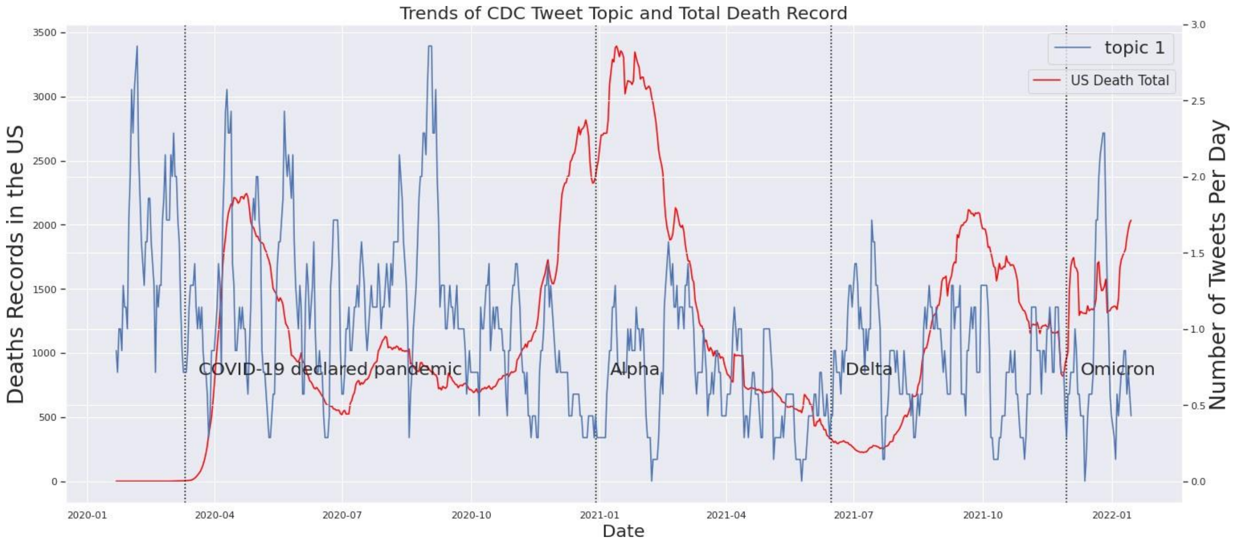

Figure 18.

- Deaths and Topic 2:

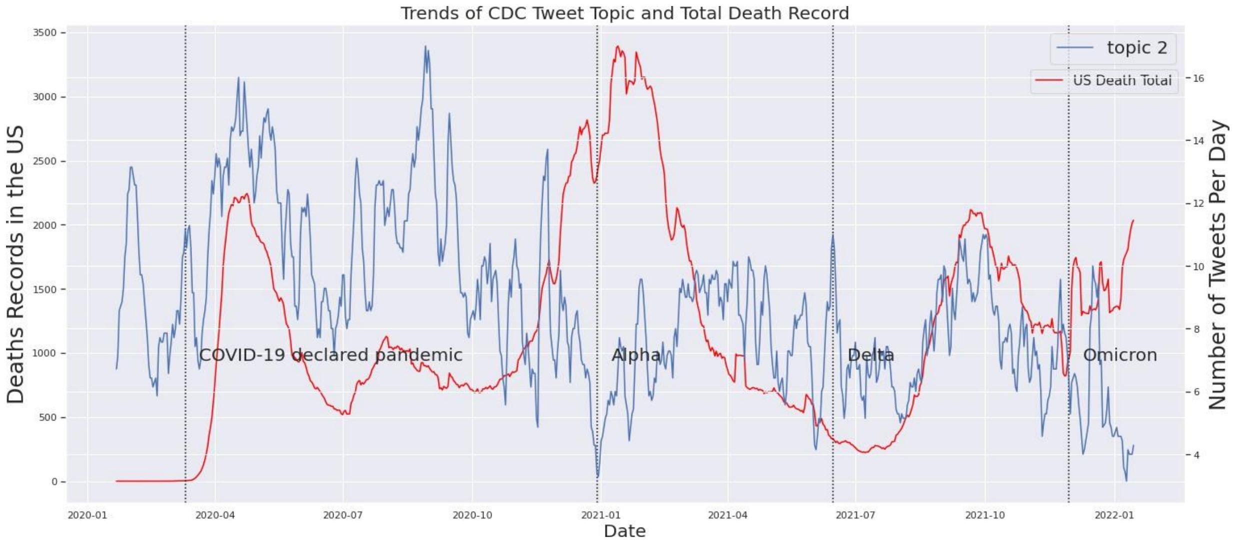

Figure 19.

- Deaths and Topic 3:

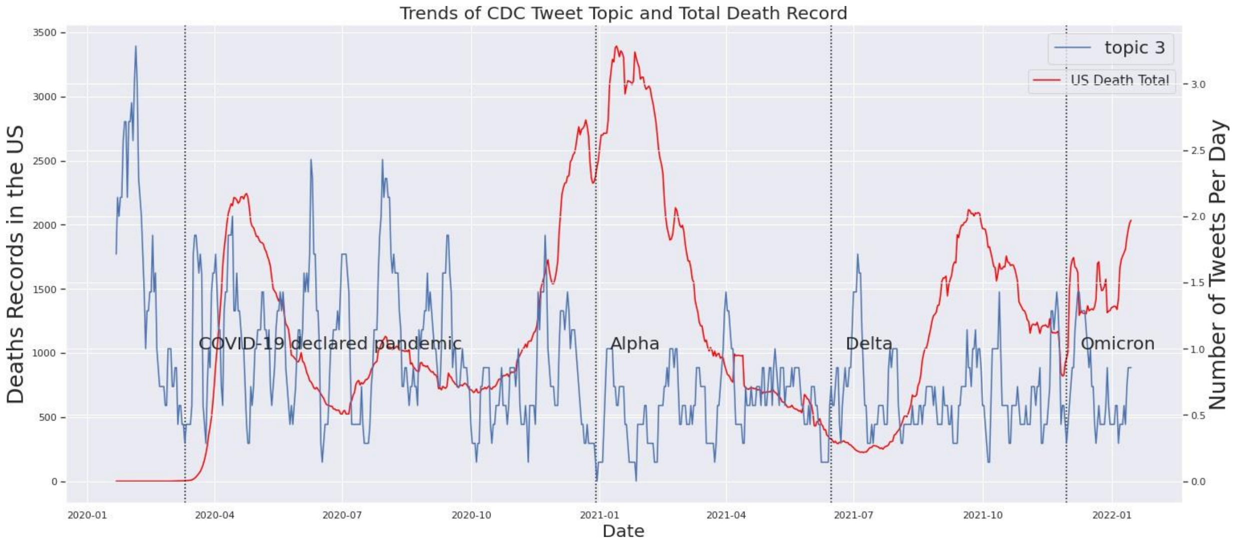

Figure 20.

- Deaths and Topic 4:

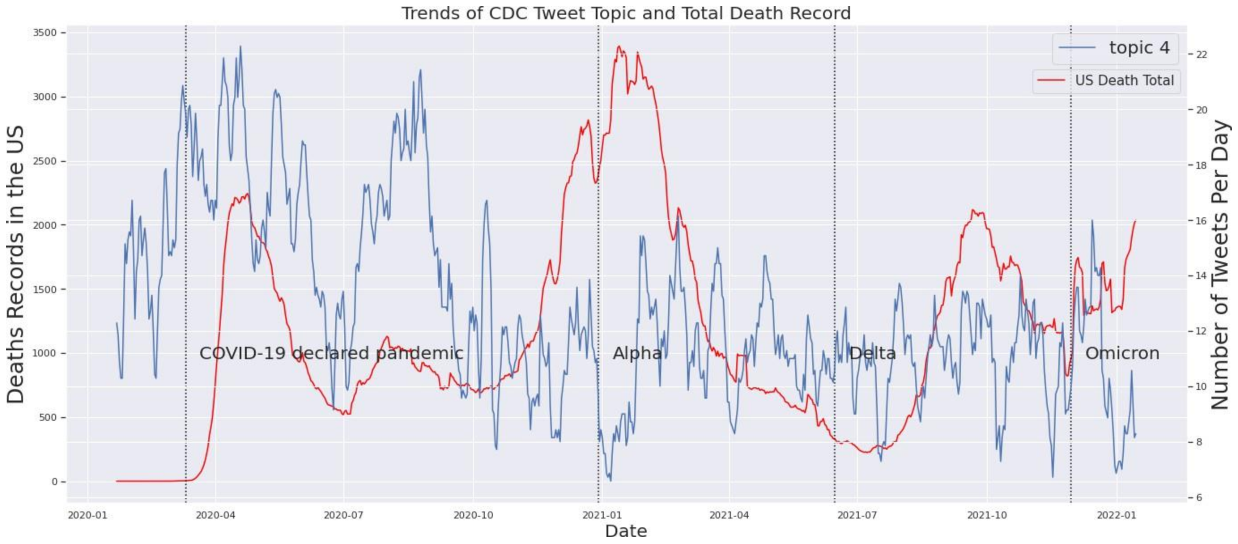

Figure 21.

- Tests and Topic 1:

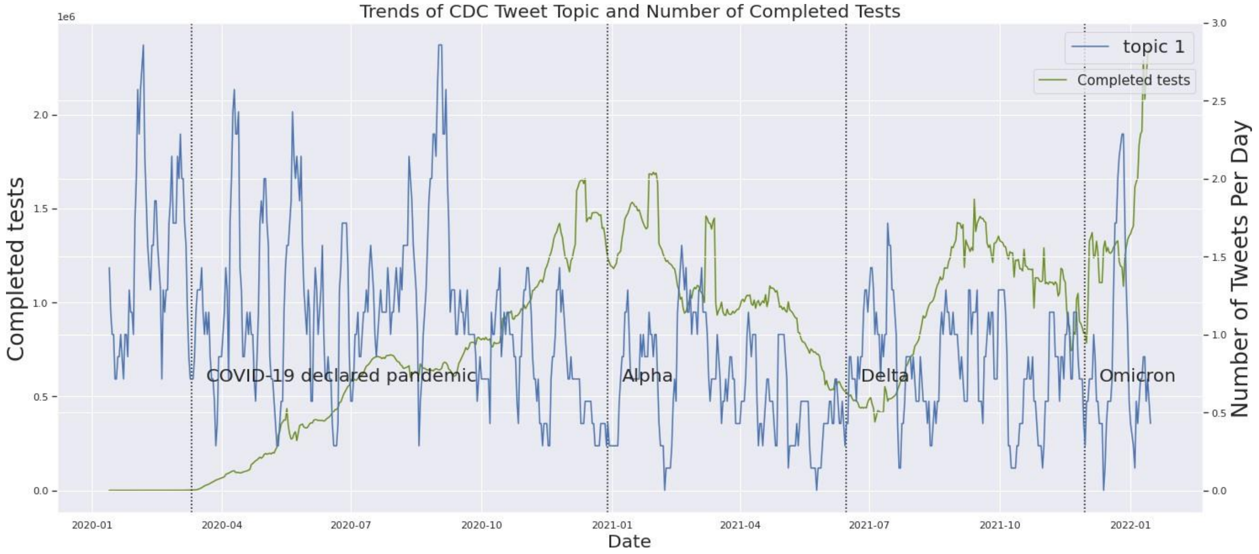

Figure 22.

- Tests and Topic 2:

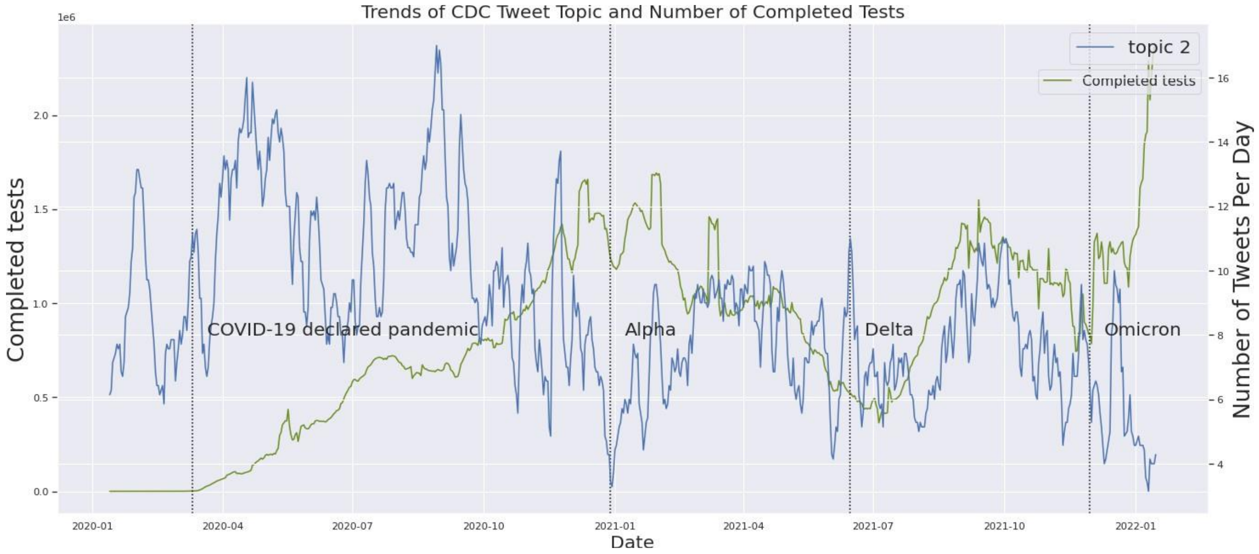

Figure 23.

- Tests and Topic 3:

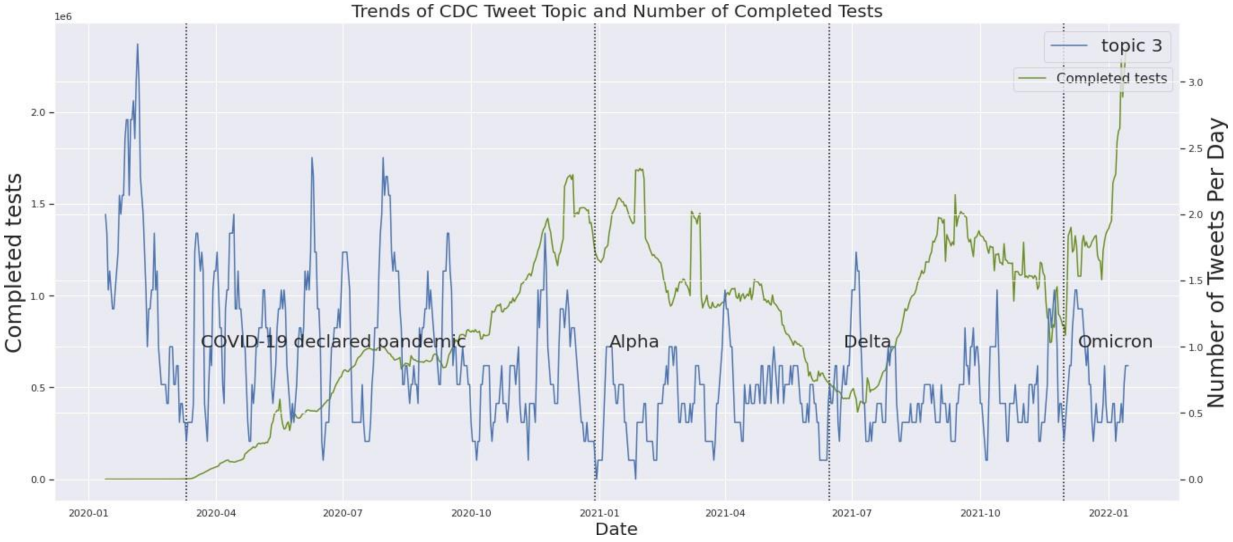

Figure 24.

- Tests and Topic 4:

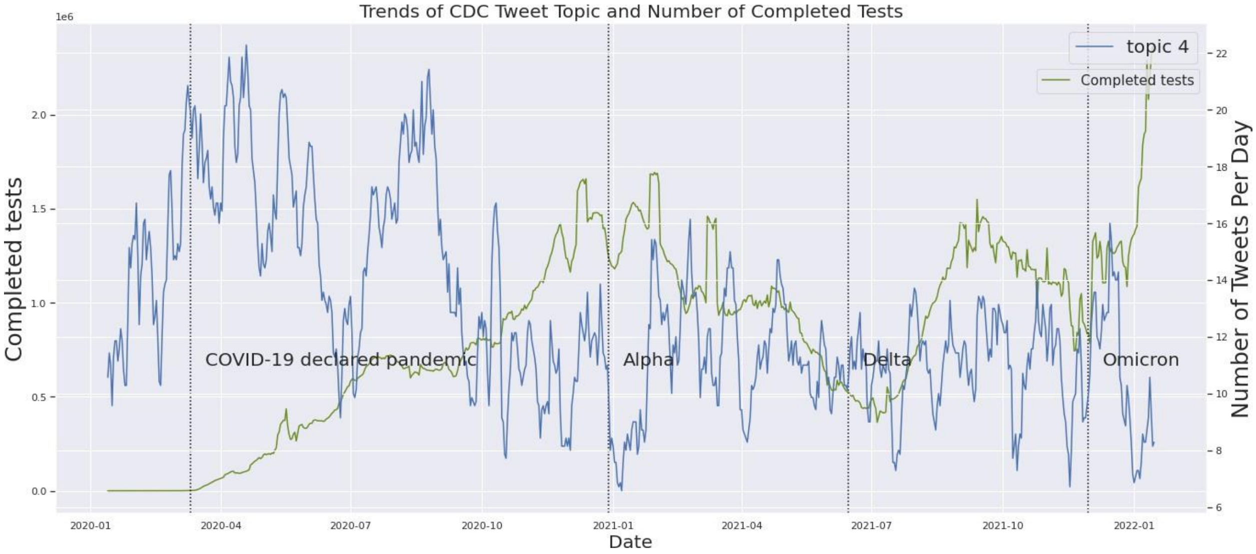

Figure 25.

- Vaccination and Topic 1:

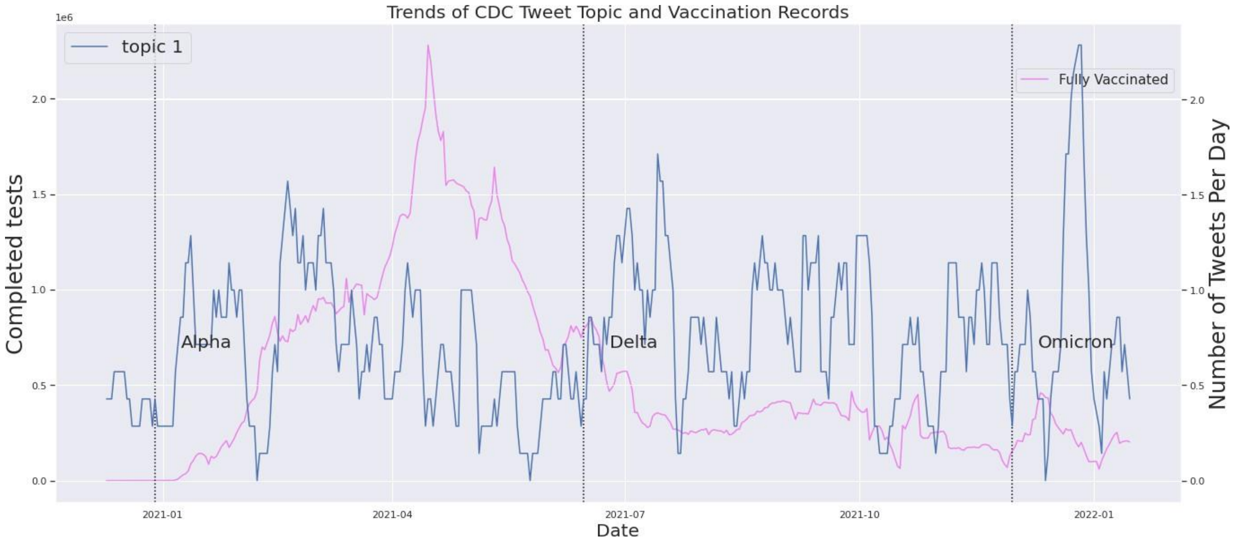

Figure 26.

- Vaccination and Topic 2:

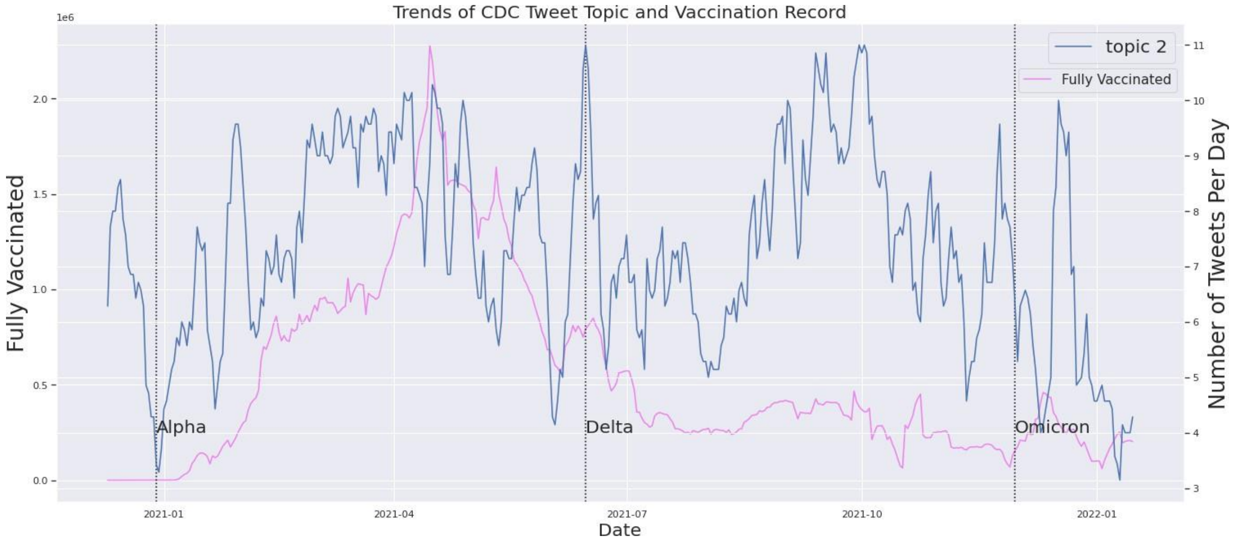

Figure 27.

- Vaccination and Topic 3

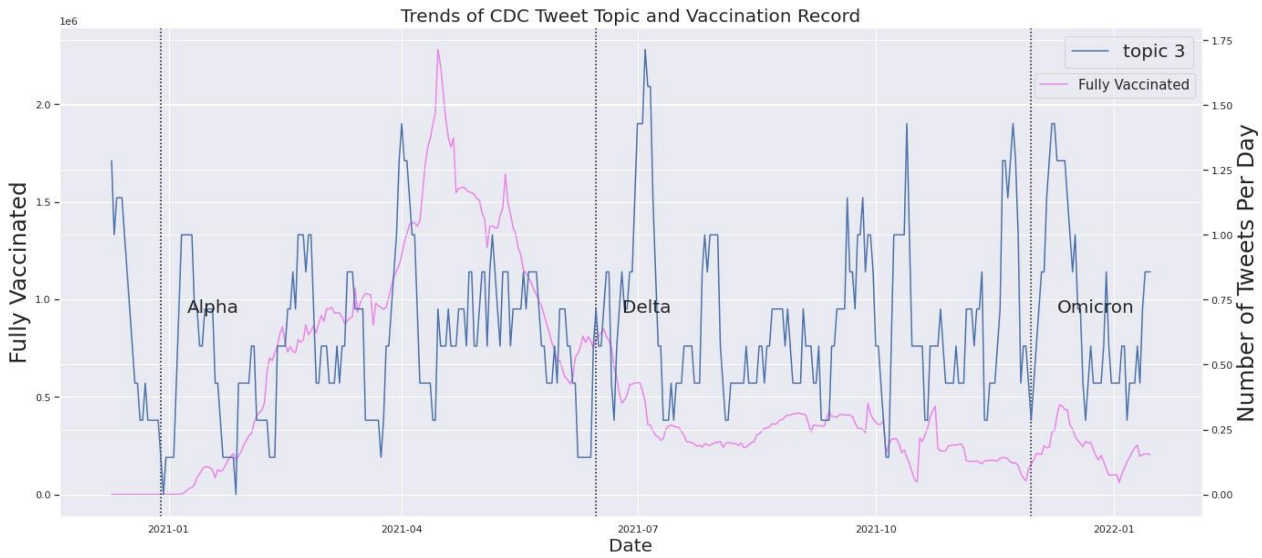

Figure 28.

- Vaccination and Topic 4:

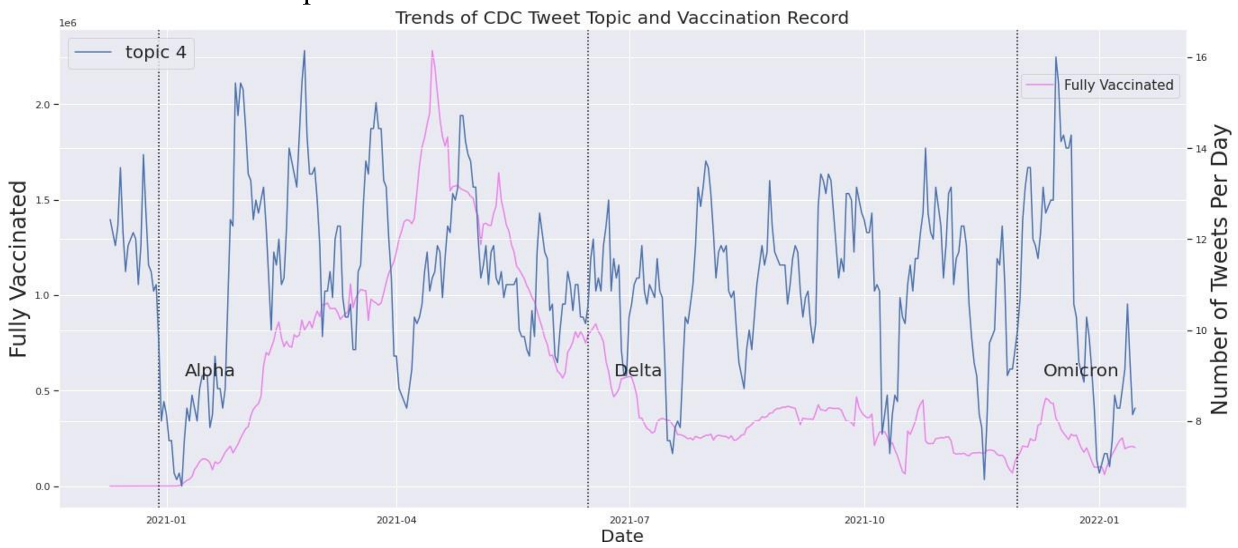

Figure 29.

Cross correlation function (CCF) plots: CDC topics and COVID-19 epidemic measurements

- Cases and topic 1

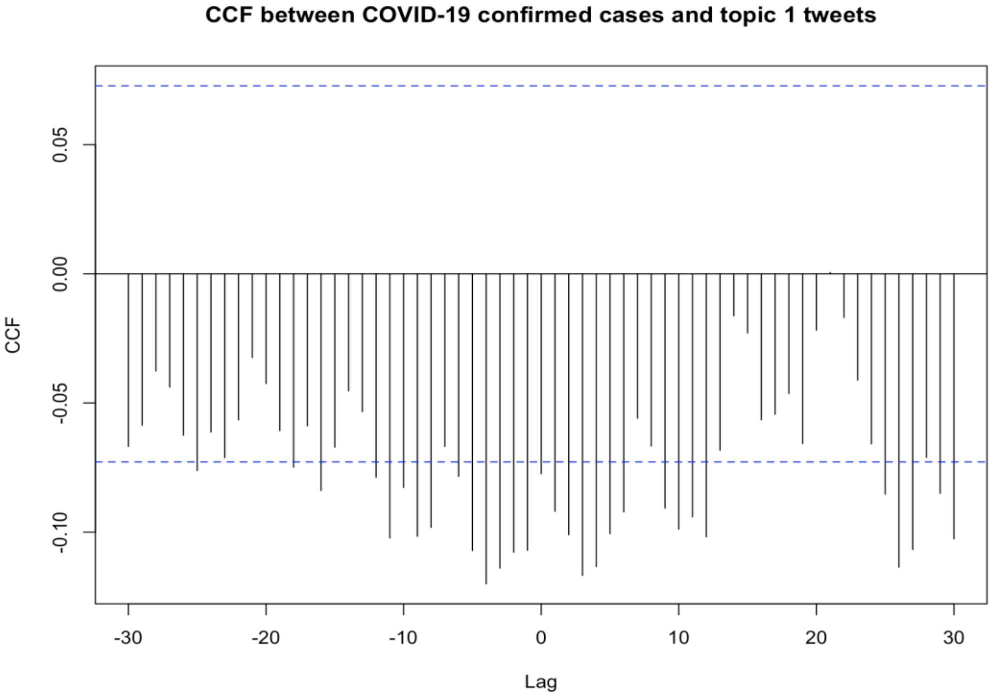

Figure 30.

- Cases and topic 2

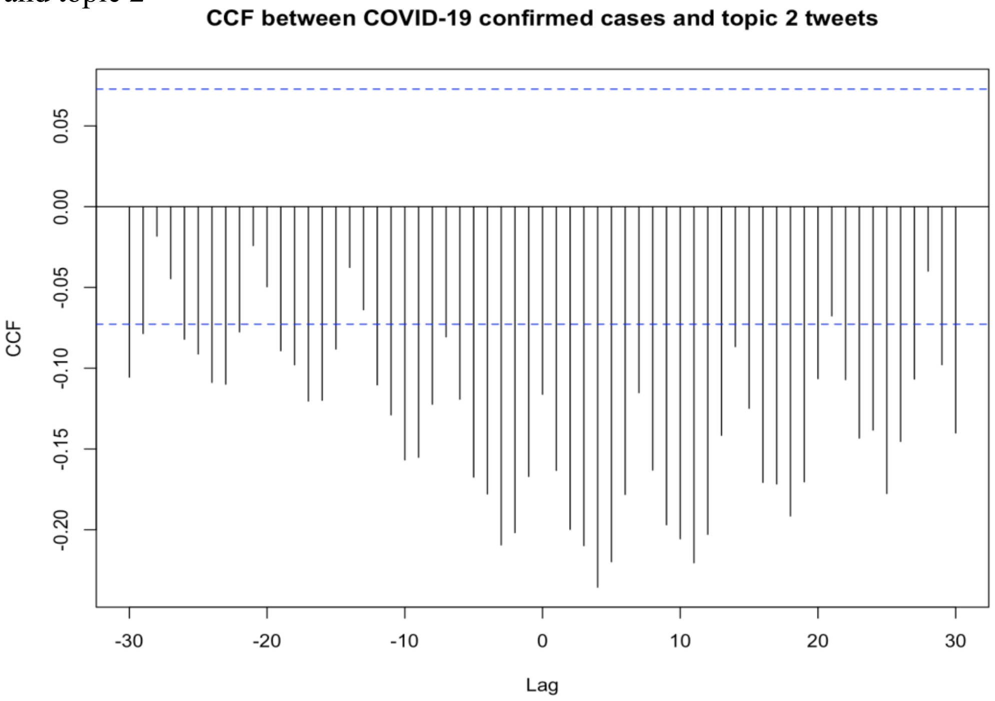

Figure 31.

- Cases and topic 3

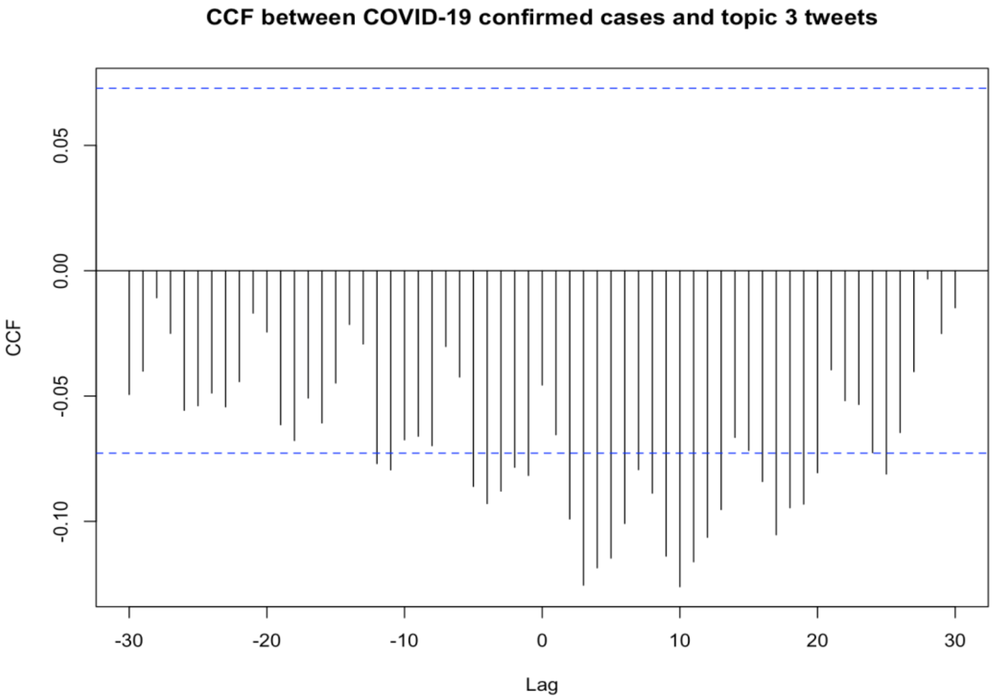

Figure 32.

- Cases and topic 4

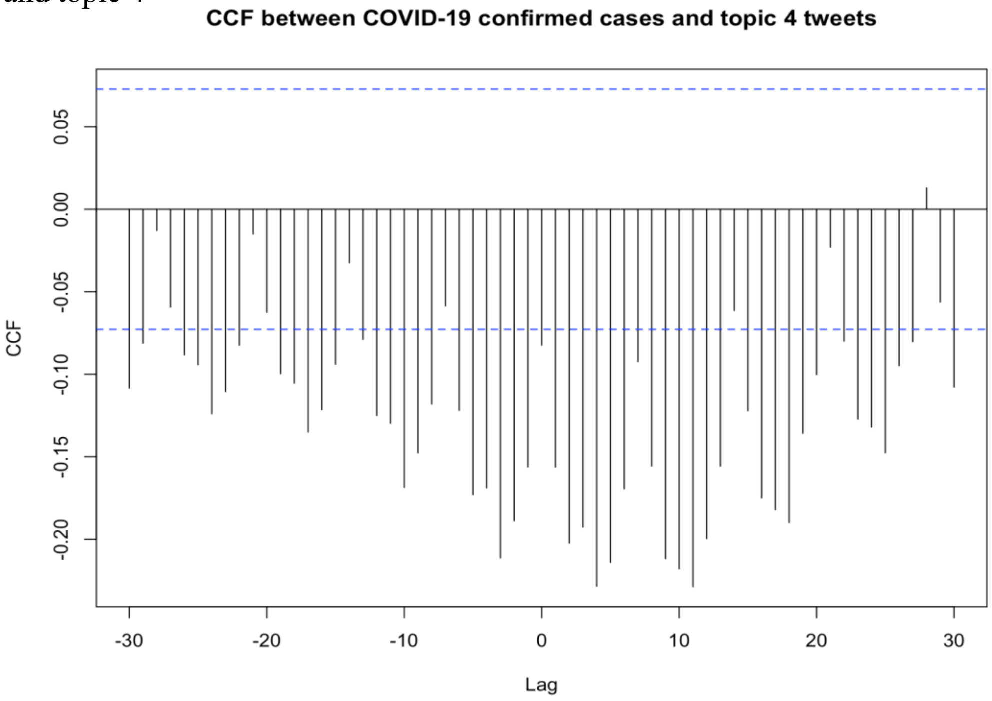

Figure 33.

- Deaths and topic 1

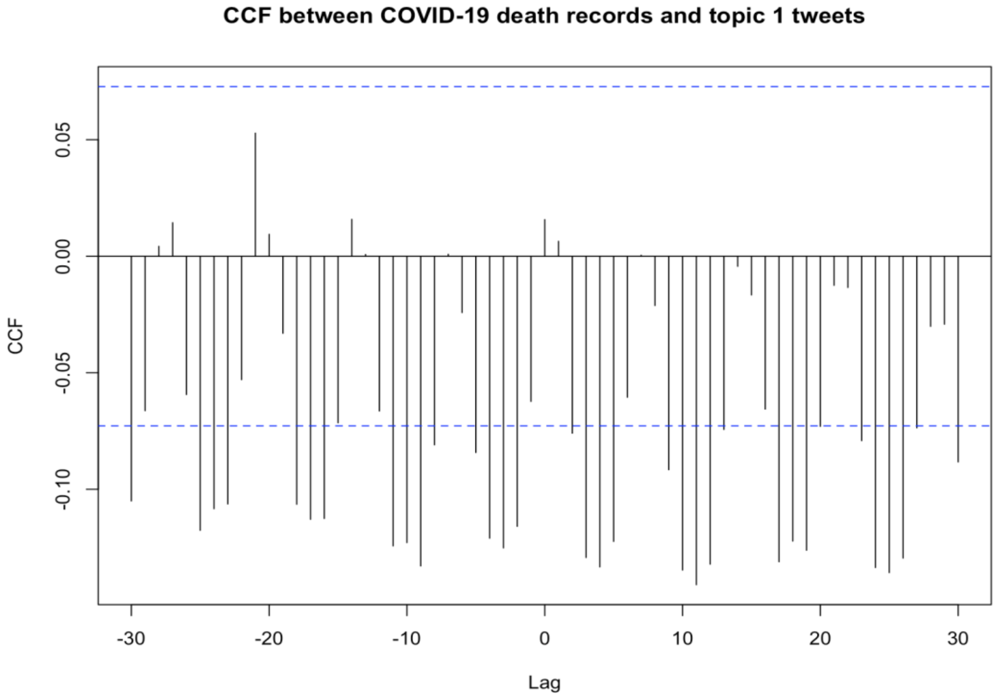

Figure 34.

- Deaths and topic 2

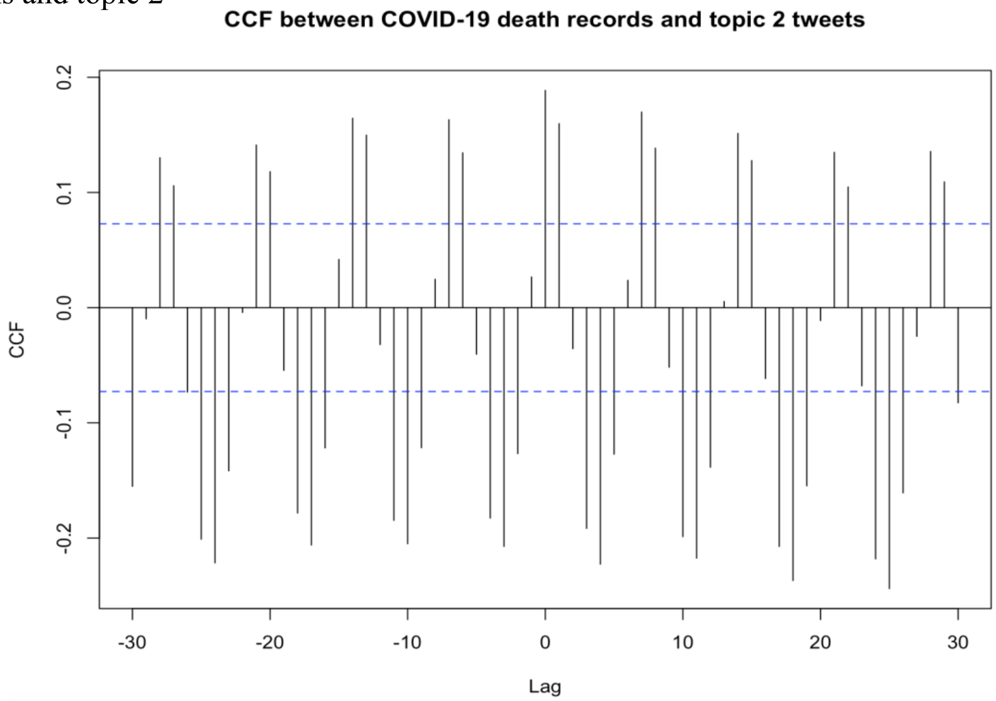

Figure 35.

- Deaths and topic 3

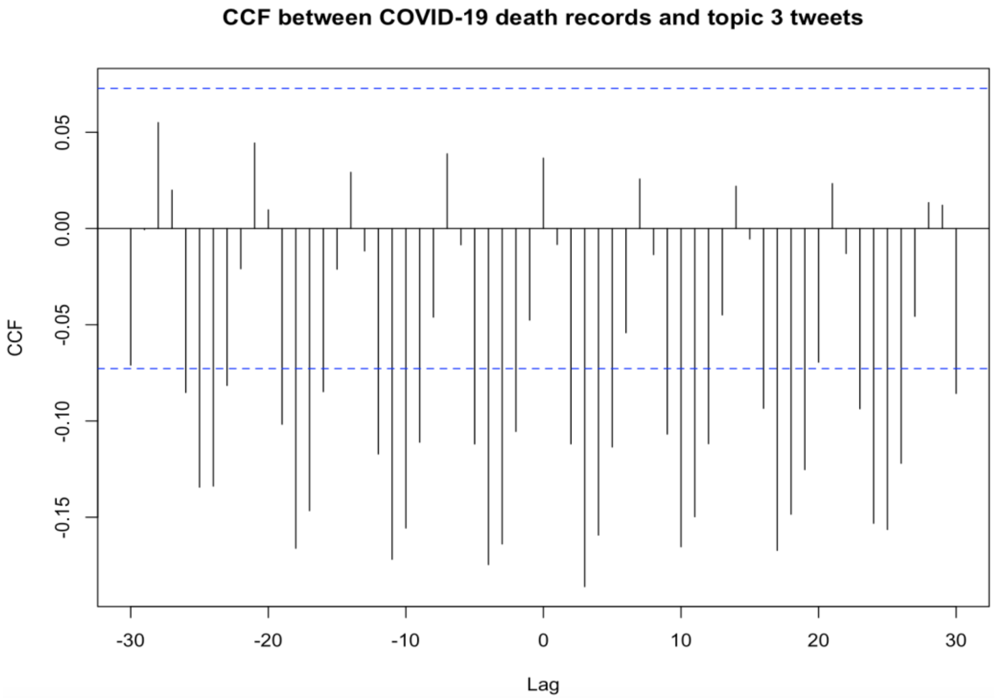

Figure 36.

- Deaths and topic 4

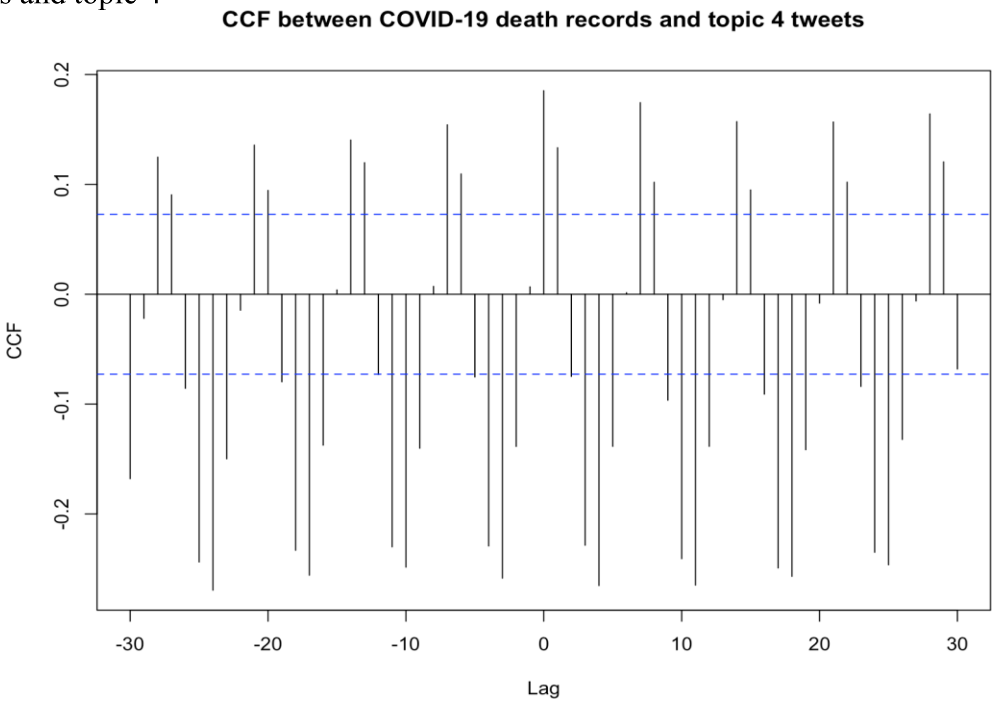

Figure 37.

- Testing and topic 1

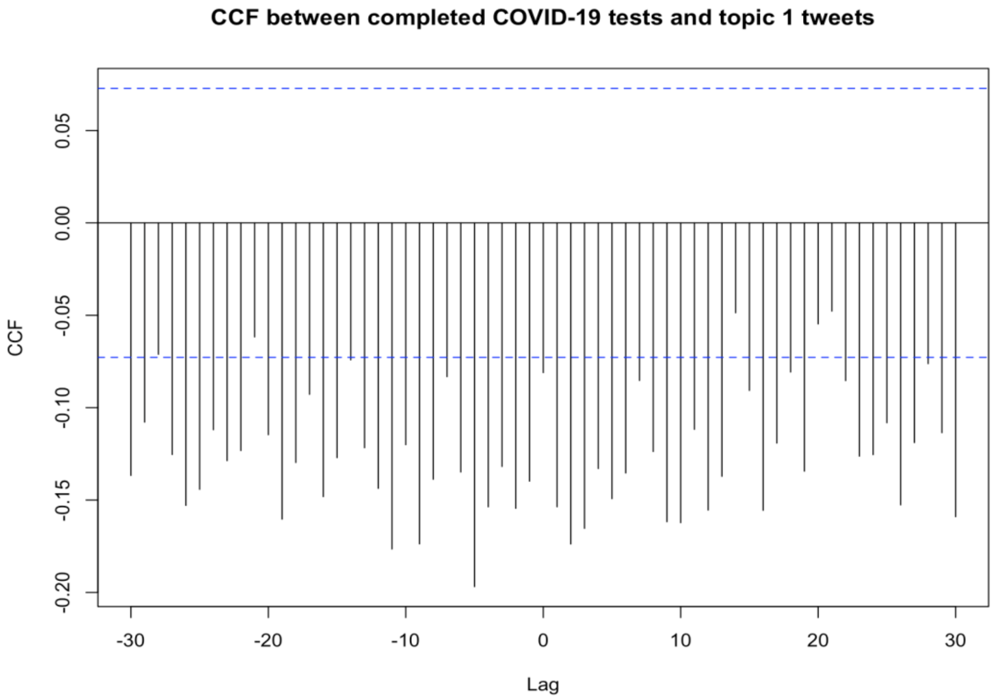

Figure 38.

- Testing and topic 2

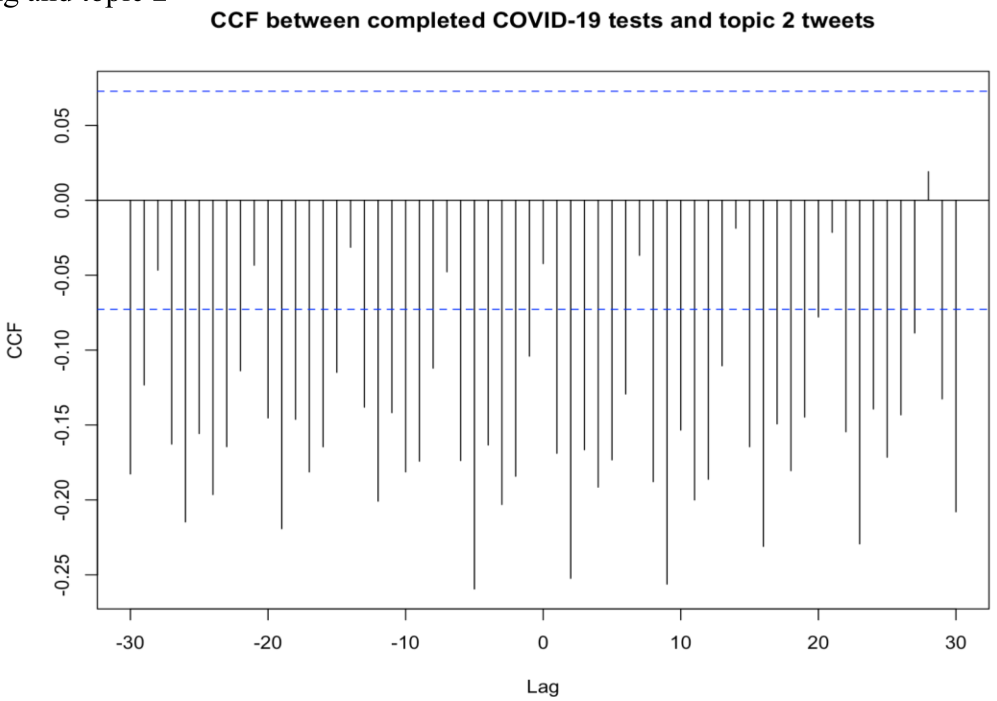

Figure 39.

- Testing and topic 3

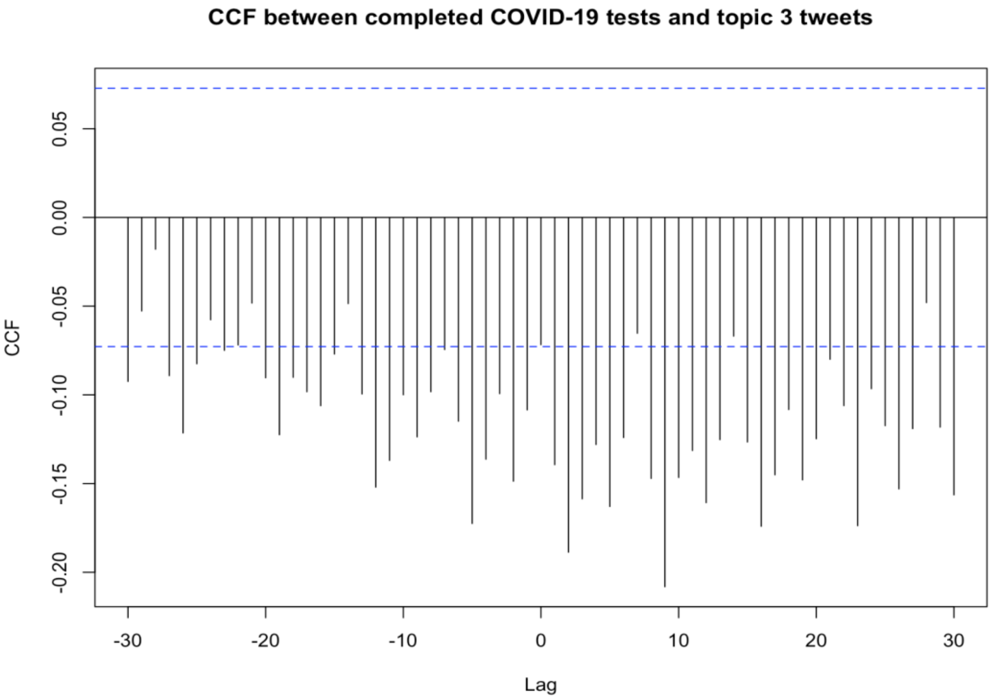

Figure 40.

- Vaccination and topic 1

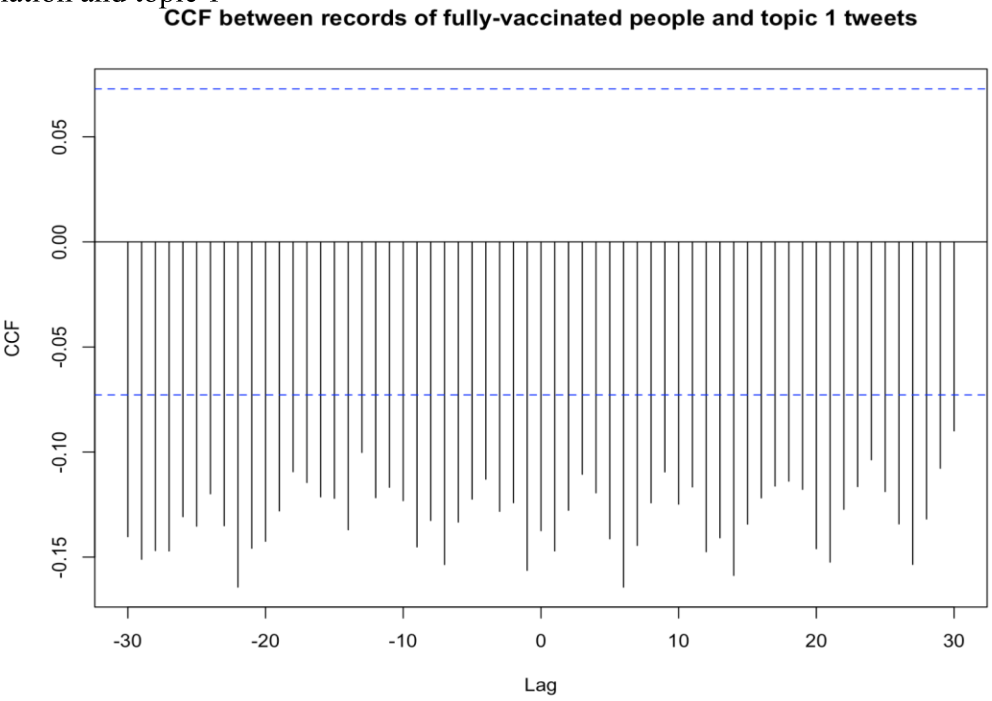

Figure 41.

- Vaccination and topic 2

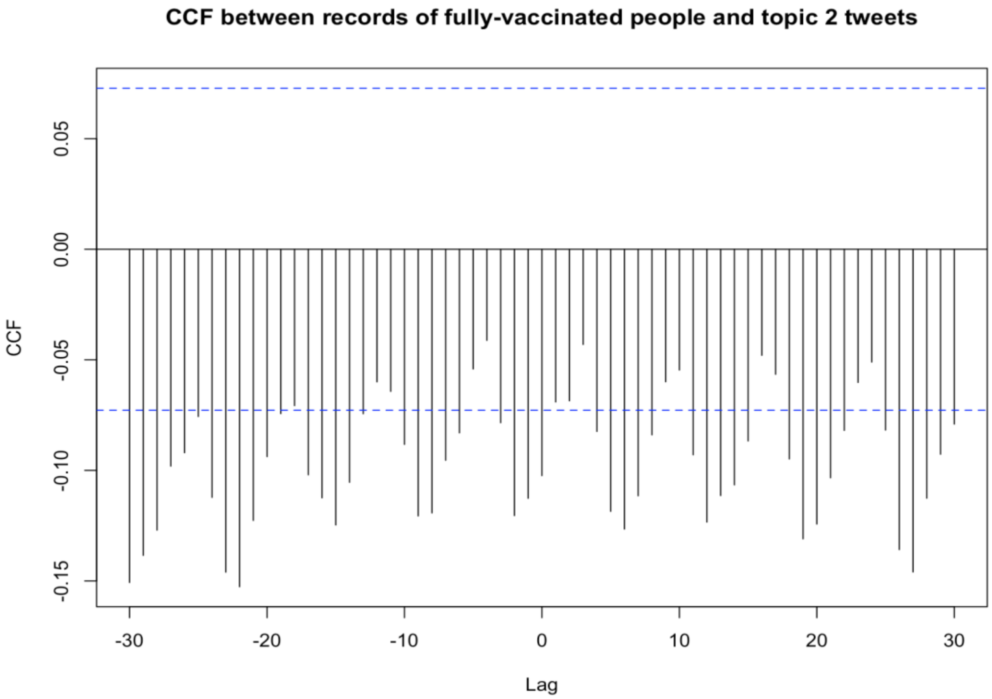

Figure 42.

- Vaccination and topic 3

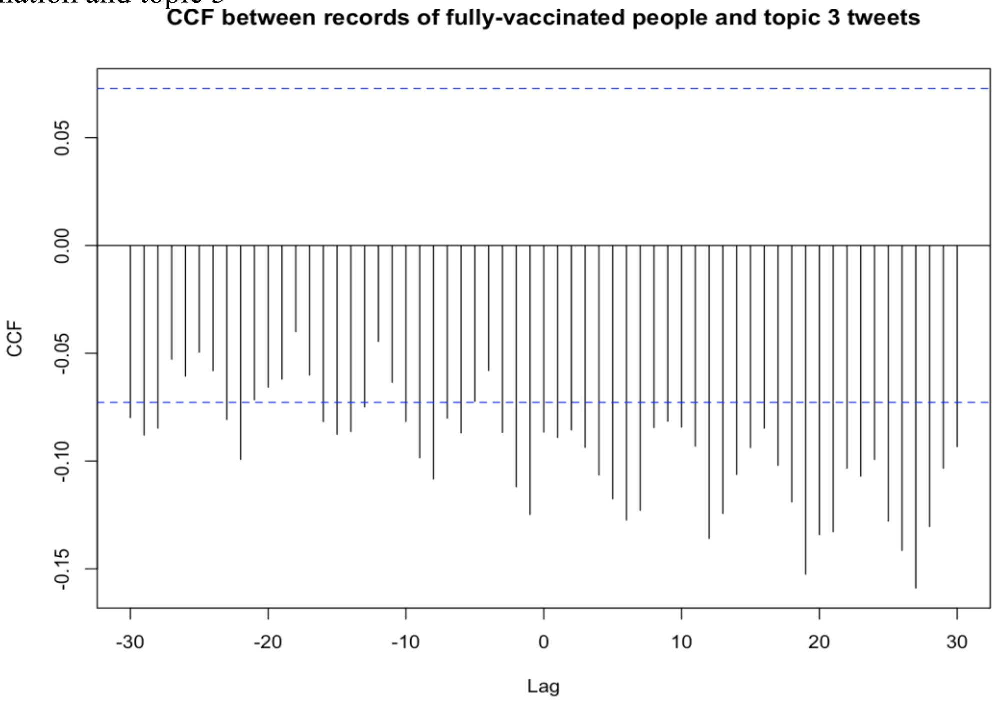

Figure 43.

- Vaccination and topic 4

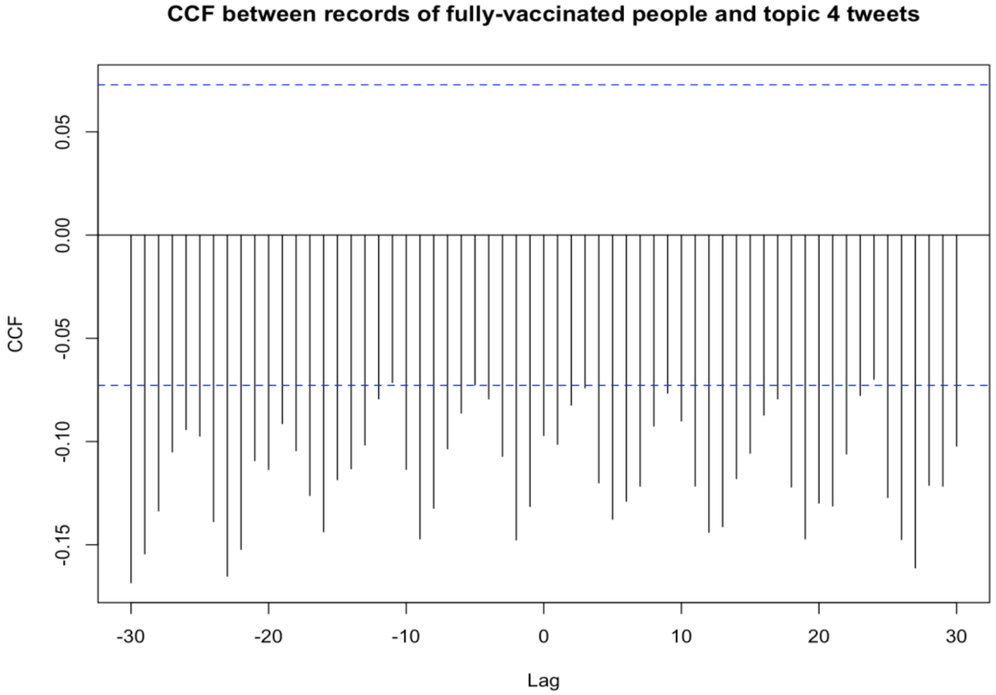

Figure 44.

Maximum and minimum cross correlation functions (CCF) values and their lags between each CDC tweet topic series and each of the four COVID-19 epidemic metrics:

Table 3. Cross correlations between CDC tweets from each topic and confirmed COVID-19 case counts in the United States from January 2020 to January 2022.

Topic	Maximum CCF	Lag at Max	Minimum CCF	Lag at Min
1	0.0005345247	21	-0.1200439	-4
2	-0.01827029	-28	-0.2356	4
3	-0.003315974	28	-0.1260986	10
4	0.01299974	28	-0.2288582	11

Table 4. Cross correlations between CDC tweets from each topic and COVID-19 death records in the United States from January 2020 to January 2022.

Topic	Maximum CCF	Lag at Max	Minimum CCF	Lag at Min
1	0.05283718	-21	-0.140962	11
2	0.1886017	31	-0.2439371	25
3	0.05504533	-28	-0.1860584	3
4	0.1853307	0	-0.2694168	-24

Table 5. Cross correlations between CDC tweets from each topic and completed COVID-19 tests in the United States from January 2020 to January 2022.

Topic	Maximum CCF	Lag at Max	Minimum CCF	Lag at Min
1	-0.04774661	21	-0.1968656	-5
2	0.01912121	28	-0.2594286	-5
3	-0.01794747	-28	-0.2081833	9

4	-0.008067961	28	-0.3283732	9

Table 6. Cross correlations between CDC tweets from each topic and fully vaccinated records for COVID-19 in the United States from January 2020 to January 2022.

Topic	Maximum CCF	Lag at Max	Minimum CCF	Lag at Min
1	-0.08994579	30	-0.1642789	6
2	-0.041227	-4	-0.1526447	-22
3	-0.03996313	-18	-0.1589435	27
4	-0.06988632	24	-0.1684011	-30

Mutual Information:

Table 7. Mutual information between each topic in the United States from January 2020 to January 2022.

Mutual Information	Topic 1	Topic 2	Topic 3	Topic 4
Topic 1	1.319797	0.1398849	0.06290801	0.1713954
Topic 2	0.1398849	3.039821	0.1801499	0.7693809
Topic 3	0.06290801	0.1801499	1.255116	0.2175483
Topic 4	0.1713954	0.7693809	0.2175483	3.338237
